# Shapeshifting Nanocatalyst for CO_2_ Conversion

**DOI:** 10.1002/adma.202509814

**Published:** 2025-09-18

**Authors:** Gustavo Zottis Girotto, Maximilian Jaugstetter, Dongwoo Kim, Lívia P. Matte, Tara P. Mishra, Mary Scott, Ruan M. Martins, André R. Muniz, Miquel Salmeron, Slavomir Nemsak, Fabiano Bernardi

**Affiliations:** ^1^ Programa de Pós‐Graduação em Física Instituto de Física Universidade Federal do Rio Grande do Sul Porto Alegre RS Brazil; ^2^ Advanced Light Source Lawrence Berkeley National Laboratory Berkeley CA USA; ^3^ Materials Science Division Lawrence Berkeley National Laboratory Berkeley CA USA; ^4^ Department of Physics and Photon Science Gwangju Institute for Science and Technology Gwangju 61005 South Korea; ^5^ Department of Chemical Engineering Universidade Federal do Rio Grande do Sul Porto Alegre RS 90040‐040 Brazil; ^6^ Department of Physics and Astronomy University of California Davis CA 95616 USA; ^7^ National Center for Electron Microscopy Molecular Foundry Lawrence Berkeley National Laboratory Berkeley CA USA; ^8^ Department of Materials Science and Engineering University of California Berkeley Berkeley CA USA

**Keywords:** artificial photosynthesis, CO_2_ reduction reaction, in situ measurements, morphology changes, photocatalysis

## Abstract

The conversion of CO_2_ into high‐value chemicals through a photoreduction reaction in water is a promising route to reduce the dependence on fossil fuels. Enhancing selectivity toward hydrocarbons or alcohols can be achieved by Ag‐Cu alloys. However, the stabilized surface state created by Ag‐Cu interactions is still poorly understood. In this work, multi‐modal in situ X‐ray experiments reveals underlying mechanisms and the evolution of Ag‐Cu nanoparticles under CO_2_ reduction reaction (CO_2_RR) conditions. Both morphological and chemical changes of Ag and Cu species induced by diffusion mechanics are tracked during nanocatalyst operation. The initial spheroid Ag‐Cu nanoparticles are composed of a Cu‐rich shell and Ag‐rich core. The reduction treatment promotes Ag migration toward the surface. During photocatalytic CO_2_ reduction reaction, Cu atoms migrate back to the surface, forming Ag‐Cu‐O species. The study observes the surface oxidation of Cu(0) to Cu^+^ and the presence of Ag at the sub‐surface region. Furthermore, nanoparticles change their shape, decreasing their specific surface area, driven by Cu diffusion during the CO_2_ photoreduction reaction. The results provide invaluable insights into the dynamic restructuring of the catalyst under reaction conditions and into the active species responsible for CO_2_ conversion.

## Graphical abstract

A multi‐modal in situ characterization of Ag‐Cu nanoparticles during CO_2_ photoreduction sheds light on dynamic transformations occurring during reaction. The Cu(core)‐Ag(shell) nanoparticles present the Ag replacement by Cu atoms. Ag is trapped at the subsurface forming a Ag‐Cu‐O phase, and the nanoparticle spreads over the substrate. These transformations are responsible for the enhanced performance of Ag‐Cu nanoparticles in the CO_2_ photoreduction.

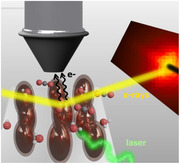

## 1 Ag‐Cu Materials as Promising Catalysts for CO_2_ Reduction Reaction

CO_2_ reduction reaction (CO_2_RR) is an ambitious strategy to overcome the current energy needs and the climate change issue.^[^ ^1^ ^]^ In the widely employed electrochemical CO_2_RR, Au‐Ag alloy catalysts are highly effective for producing CO with high current densities.^[^ ^2^, ^3^ ^]^ On the other hand, Cu surfaces have an inherent preferable intermediate binding energy to produce extended products such as hydrocarbons and alcohols.^[^ ^4^, ^5^ ^]^ Moreover, Cu surfaces are the only pure transition metal surfaces that demonstrate the ability to protonate *CO, which is a rate‐limiting step of the reaction. Thus, several studies investigated the combination of Cu and Ag to control activity and selectivity in electrochemical CO_2_RR.^[^ ^6^, ^7^, ^8^ ^]^ The generation of hydrocarbon products with Ag‐Cu alloys during electrochemical CO_2_RR is usually attributed to *CO spillover, which is first generated in Ag and then diffuses to the Cu atoms present in the surface, where multi‐electron processes take place, leading to the possibility of C‐C coupling.^[^ ^9^ ^]^

Another possibility of CO_2_RR is to combine CO_2_ and H_2_O using solar light to drive the photocatalytic CO_2_RR, which is also known as artificial photosynthesis.^[^ ^10^ ^]^ Considering photocatalytic CO_2_RR, metals and metal oxides have been investigated in the reaction to produce alcohols and hydrocarbons.^[^ ^10^, ^11^, ^12^ ^]^ In particular, it has been demonstrated that Au^[^ ^13^ ^]^ and Ag^[^ ^14^ ^]^ nanoparticles can initiate CO_2_ reduction using H_2_O as a proton source under only visible light exposure. However, noble metals are active but not selective toward hydrocarbons or alcohols for this reaction.^[^ ^14^ ^]^ Cu can be added to enhance selectivity, despite its low activity in artificial photosynthesis.^[^ ^15^ ^]^ The literature reports some interesting results for photocatalytic CO_2_RR using Ag‐Cu‐based systems. Vahidzadeh et al studied Ag‐Cu bimetallic nanoparticles supported on TiO_2_ nanotube arrays in the photocatalytic CO_2_RR.^[^ ^16^ ^]^ The system demonstrated preferred formation of ethane in the reaction, which was attributed to the facilitated C‐C coupling due to an asymmetric charge distribution existing from the presence of adjacent adsorption sites. Ag‐Cu bimetallic nanoparticles with different Ag/Cu ratios were also used, encapsulated by UiO_66_‐NH_2_ MOF to avoid aggregation of the nanoparticles.^[^ ^17^ ^]^ These nanoparticles presented great activity, cycling stability, and long‐term stability with the formation of C_1_ or C_2_ products depending on the Ag/Cu ratio chosen. There are other reports using different Ag‐Cu structures for photocatalytic CO_2_RR. For example, Ag‐Cu sub‐nanoclusters were studied in the photocatalytic CO_2_RR, and it was obtained a record C_2_H_4_ formation rate.^[^ ^18^ ^]^ The authors observed that Ag is responsible for the C‐C coupling while Cu allows the C_2_H_4_* desorption. Cu/Cu_2_O and Ag individual nanoparticles were deposited on TiO_2_ making a solid state Z scheme heterostructure.^[^ ^19^ ^]^ In this case, there is no formation of bimetallic Ag‐Cu structures, but the system was able to produce CH_4_ with high activity and selectivity. Finally, Ag_2_Cu_2_O_3_ nanowires with abundant Cu‐Ag Lewis acid‐base dual sites at the {100} surface were used in the photocatalytic CO_2_RR and demonstrated great selectivity toward transformation of CO_2_ into CH_4_.^[^ ^20^ ^]^ Overall, the Ag‐Cu system already demonstrated promising results in CO_2_RR, but the studies are quite recent, and many open questions exist, like those related to the atomic events occurring during photocatalytic CO_2_RR.

Cu presents a high adsorption energy to intermediate products such as *COOH, which can be transferred to regions of low concentration of adsorbed species, such as the Ag surface.^[^ ^21^ ^]^ Increasing the Ag concentration in these alloys lowers the average binding energy of adsorbed *CO species.^[^ ^22^ ^]^ Despite the low *CO concentration in Ag sites, the incorporation of Ag single atoms into Cu nanoparticles strengthens the CO bond at adjacent Cu atoms due to the presence of compressive strain and d‐band center modification.^[^ ^23^ ^]^ It has also been observed that the selectivity for CO_2_RR is improved by increasing the Ag‐Cu interfacial area.^[^ ^21^ ^]^ Charge transfer was suggested to strengthen the adsorption of *CO intermediates on Cu sites after CO_2_ adsorption on Ag sites,^[^ ^24^ ^]^ and electron‐deficient Cu sites are hypothesized to provide adsorption sites for alcohol product intermediates.^[^ ^25^ ^]^ Regarding the atomic configuration evolution of Ag‐Cu nanoparticles during CO_2_RR, phase separation of Ag‐Cu occurs during electrochemical CO_2_RR when Cu suffers reduction by cathodic currents.^[^ ^26^ ^]^ Moreover, a stabilized Cu^+^ overlayer was also found to be induced by Ag presence during electrochemical CO_2_RR with electrodeposited Ag‐Cu alloys.^[^ ^27^ ^]^ Lastly, the oxidation state and morphology of Cu nanoparticles evolve during photoreaction due to photocorrosion,^[^ ^28^ ^]^ where either oxidation or reduction processes occur. The mixing with Ag leads to another complexity in this regard, and there is a poor understanding about possible changes in the atomic arrangement and morphology of Ag‐Cu nanostructures during photocatalytical CO_2_RR.

All these competing and synergistic processes lead to high complexity of this system under operating conditions. Determining the surface atomic population and nanoparticle morphology during reaction conditions is therefore of utmost importance to understand underlying processes and to further predict the correct reaction mechanisms. Consequently, it allows the design of future improved photocatalysts for CO_2_RR. To study both chemical and morphological transformations of the Ag‐Cu catalyst on the atomic level in situ, we applied a multi‐modal soft X‐ray characterization together with Molecular Dynamics simulations to elucidate the behavior of Ag‐Cu nanoparticles during photochemical CO_2_RR.

## 2 Ex Situ Characterization

**Figure 1:**
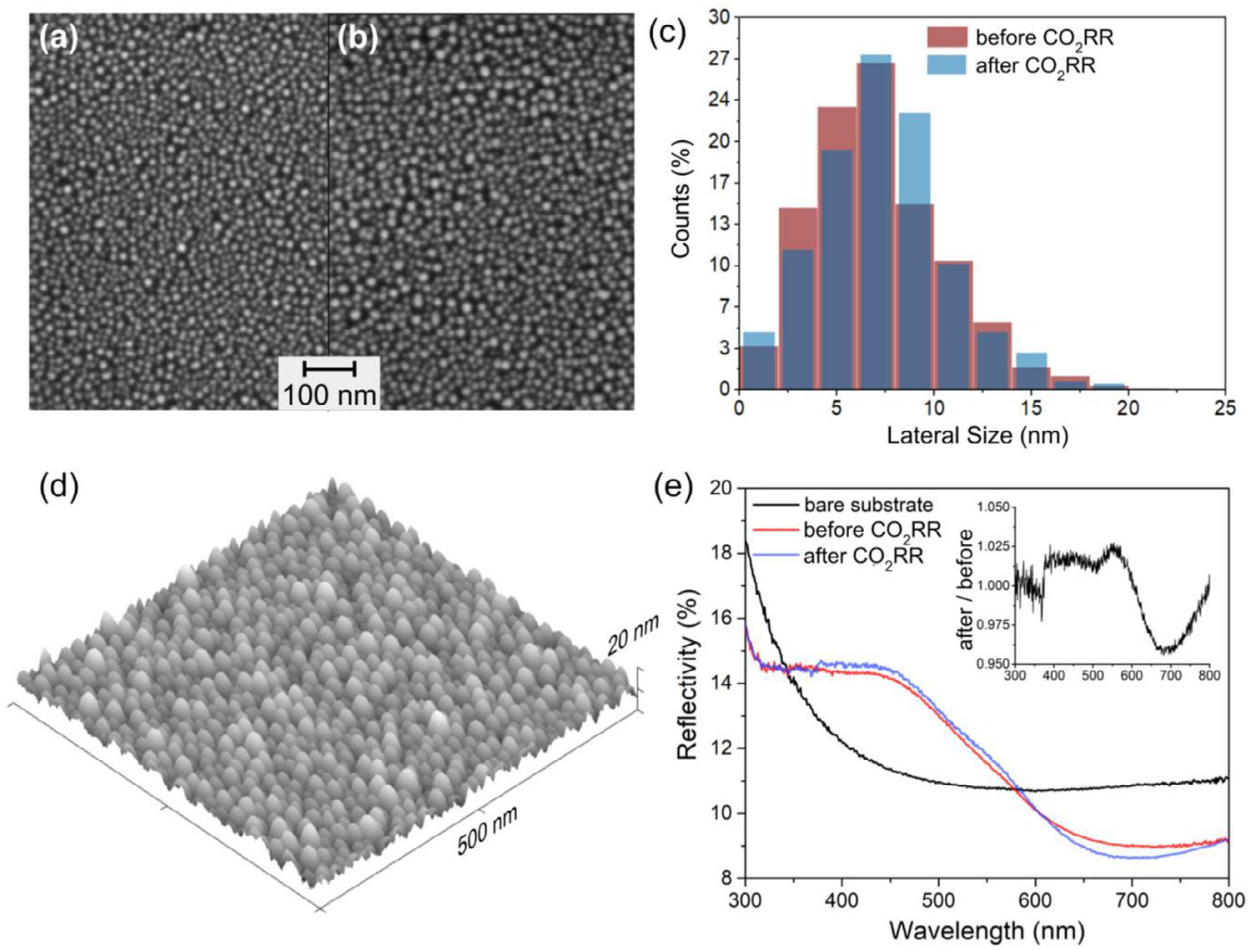
Typical SEM images of AgCu nanoparticles a) before and b) after exposure to CO_2_RR. c) Histogram of nanoparticle lateral width before and after exposure to CO_2_RR. d) Typical AFM image obtained of the sample after exposure to CO_2_RR. e) UV–vis spectroscopy measured in total reflectance mode before and after exposure to CO_2_RR. The inset shows the ratio between the spectra of the sample after and before exposure.

A typical SEM image of the Ag‐Cu nanoparticles before and after exposure to CO_2_RR conditions is displayed in **Figure** **1**a,b. The as‐prepared Ag‐Cu nanoparticles present a lateral mean size ≈7 nm. SEM measurements confirm that nanoparticles’ lateral dimensions slightly increase after CO_2_RR (Figure 1c). The analysis of AFM images (Figure 1d; Figure S1, Supporting Information) demonstrates that the particles show slight elongation in the direction normal to the surface. The UV‐Vis spectra (Figure 1e) of the Ag‐Cu nanoparticles show an increase in background as compared to the bare Si case. The increase in reflectance centered at 450 nm evidences the existence of Local Surface Plasmon Resonance (LSPR) due to Ag nanoparticles, which may promote the generation of hot carriers^[^ ^29^ ^]^ or increase the electromagnetic near‐field^[^ ^30^ ^]^ that induces CO_2_RR.^[^ ^31^ ^]^ After exposure to CO_2_RR, a new oscillation ≈550 nm arises, thus close to the wavelength of the laser irradiated to activate the reaction (532 nm), which is consistent with some structural modification taking place.

Figure S2 (Supporting Information) shows the FTIR measurements of the CO_2_RR products of Ag‐Cu/Si nanoparticles. Initially, the presence of the band at ≈1007 cm^−1^ is most likely connected to carbonate species present in the liquid after desorption from the surface. After CO_2_RR, this band is modified and matches exactly that from a methanol standard, thus evidencing the methanol formation over the Ag‐Cu/Si nanoparticles studied. One could argue whether this new band is from carbonate as well, so GC‐FID measurements were conducted. Figure S3 (Supporting Information) shows the GC‐FID results where the methanol formation is clearly identified in the solution after CO_2_RR through comparison with pure methanol standard. Ag‐Cu/TiO_2_ nanoparticles were synthesized through the chemical route and present the same atomic structure and similar size as the Ag‐Cu/Si nanoparticles synthesized through the thermal evaporation method (see Figures S4–S7, Supporting Information, and later discussion of Ag‐Cu/Si nanoparticles in the main text). These powder catalysts, which are closer to their applicable counterparts, also present the formation of methanol as observed in Figure S8 (Supporting Information). Therefore, Ag‐Cu/Si nanoparticles are a relevant system to be investigated during photocatalytic CO_2_RR.

## 3 Probing Changes in the Surface Atomic Configuration

**Figure 2:**
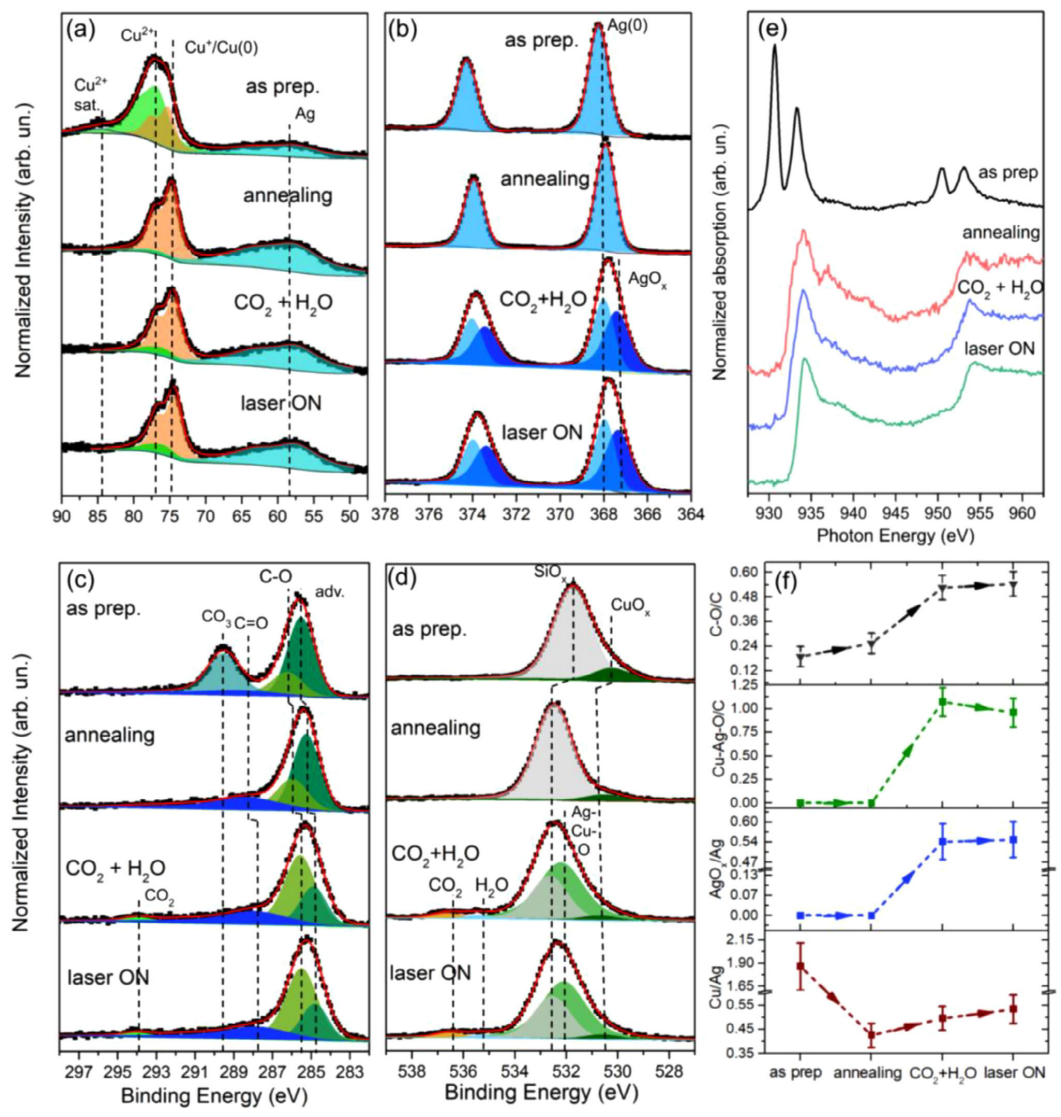
AP‐XPS spectra at a) Cu 3p + Ag 4p, b) Ag 3d, c) C 1s, and d) O 1s electronic regions in the as‐prepared, after annealing at 200 °C in 20 mTorr H_2_, during CO_2_RR with 40 mTorr CO_2_ + 40 mTorr H_2_O with laser off, and during 532 nm laser on conditions. e) XAS measurements at Cu L edge measured in drain‐current mode in the same conditions. f) correlations taken out of AP‐XPS measurements between Cu/Ag, AgO_x_/Ag, Cu‐Ag‐O/C, and C‐O/C ratio (atomic %) on the surface.

The AP‐XPS measurements at Cu 3p, Ag 4p, Ag 3d, C 1s, and O 1s regions using 695 eV excitation energy are displayed in **Figure** **2**a–d. The Cu 3p XPS region shows contributions from Cu^+^/Cu(0) and Cu^2+[^ ^32^ ^]^ with an energy separation of 1.5 eV, and similar components are observed using 1240 eV photon energy (see SI for further discussion). The high concentration of Cu^2+^ at the surface is expected since the sample has been exposed to air and humidity before being introduced to the experimental chamber. Only metallic Ag(0) is found at Ag 3d XPS region of the as‐prepared sample.^[^ ^33^ ^]^ The C 1s XPS region exhibits a large concentration of carbonate (CO_3_^2−^) species at binding energy ≈289.5 eV,^[^ ^34^ ^]^ originating from the contamination during the growth in the evaporation chamber and the subsequent air exposure. The O 1s XPS region presents two components associated to SiO_x_ and CuO_x_ (convolution of Cu^2+^ and Cu^+^ species)^[^ ^35^ ^]^ related to the substrate and Ag‐Cu nanoparticles, respectively. The annealing process at 200 °C removes the CO_3_^2−^ component in the C 1s region, and the Cu^2+^ component almost disappears, indicating that Cu has reduced to either Cu^+^ or Cu(0). This observation correlates with the decrease of CuO_x_‐related components in the O 1s region. The normalized Cu/Ag ratio obtained using the Cu 3p and Ag 4p areas is 1.8, and the one obtained with 1240 eV photon energy is ≈1.6 (Figure S9, Supporting Information). Simulation with the Electron Spectra for Surface Analysis (SESSA)^[^ ^36^ ^]^ software gives a spheroid with a 6–7 nm Ag core diameter covered by a 1 nm CuO shell (Figure S10, Supporting Information). Thus, the representative Ag‐Cu stoichiometry is approximated as Ag1Cu1. Figure S11 (Supporting Information) shows STEM‐EELS measurements of the as‐prepared sample in agreement with this observation.

In situ XAS spectra measured during the different conditions at Cu L_3_ edge are presented in Figure 2e. The as‐prepared condition shows two different features: the one around 930 eV is expected due to the Cu^2+^ species at the surface, while the one near 934 eV corresponds to the Cu^+^ species.^[^ ^37^, ^38^ ^]^ These two features agree qualitatively with AP‐XPS measurements. After annealing, the Cu^2+^ feature disappears, and the oscillation at ≈937 eV evidences the appearance of a Cu(0) component,^[^ ^38^ ^]^ which could not be resolved with AP‐XPS only due to binding energy overlap of Cu^+^ and Cu(0) components. After dosing CO_2_ + H_2_O, the shoulder at 937 eV decreases in intensity, and the white line narrows, evidencing Cu(0) oxidation to Cu^+^. Cu oxidation starts before laser irradiation, but the incidence of visible light further accelerates the process.

Figure 2f displays how four distinct parameters change after each exposure condition. A dramatic decrease of the normalized Cu 3p/Ag 4p intensity ratio from 1.8 to 0.4 during H_2_ exposure implies that the concentration of Ag atoms at the surface increases. The subsequent introduction of a 40 mTorr CO_2_ + 40 mTorr H_2_O atmosphere at RT results in a new low‐binding energy component in the Ag 3d region, interpreted as AgO_x_.^[^ ^33^ ^]^ Furthermore, another component in the O 1s region emerges at ≈532 eV. This component is linked to the oxidation of Ag, and it is also observed a relative increase of the Cu/Ag ratio. These results point to the existence of interfacial Cu‐Ag─O chemical species.^[^ ^39^ ^]^ The dissociation of CO_2_ at the surface generates O and C─O species (evidenced by C─O component increase in C 1s spectra). Tables S1–S4 (Supporting Information) show the parameters obtained from the AP‐XPS analysis of spectra in Figure 2. Figure S12 (Supporting Information) shows the Cu 2p_3/2_ and Ag 3d quasi in‐situ XPS data of the Cu‐Ag/Si nanoparticles in the as‐prepared and after CO_2_RR state using real reaction conditions, whose results are consistent with those shown in Figure 2.

The influence of individual CO_2_ and H_2_O atmospheres in the surface states was studied by inserting 40 mTorr CO_2_ before the introduction of 40 mTorr CO_2_ + 40 mTorr H_2_O atmosphere. Figure S13 (Supporting Information) shows the comparison of the AP‐XPS spectra. In the Cu 3p region, it is possible to observe a slight oxidation to Cu^2+^ after CO_2_ dosing, and it is further oxidized with the later insertion of H_2_O. In the Ag 3d electronic region, CO_2_ begins the oxidation of Ag(0) to AgO_x_ but again, the introduction of H_2_O increases it. At the C 1s and O 1s electronic regions, the C‐O and Ag‐Cu‐O components, respectively, increase after CO_2_ introduction, and this increase is enhanced with the CO_2_ + H_2_O atmosphere. Overall, the main components coming up in the CO_2_RR are already present with the CO_2_ atmosphere, but the insertion of H_2_O increases it even more, so its formation occurs as a part of the photocatalytic reaction.

The Cu/Ag ratio increases after dosing CO_2_ and H_2_O. Although the AgO_x_ percentage increases with CO_2_ and H_2_O dosing, it does not change after laser irradiation. The Cu‐Ag‐O component decreases after laser irradiation. The C‐O concentration increases after every condition. Therefore, the laser triggers substitution of surface Ag by the Cu atoms, as indicated by the decrease of the Cu‐Ag‐O signal, which suggests Ag‐Cu bond breaking. However, the constant AgO_x_/Ag ratio evidences that Ag is not further oxidized by this last condition. Similar behavior was found with 1240 eV excitation energy (Figures S14 and S15, Supporting Information), and for a Ag‐Cu powder system synthesized through a precipitation method (Figures S6 and S7, Supporting Information). It is interesting to point out that similar behavior is found by annealing the sample in an O_2_ atmosphere (Figure S16, Supporting Information), which is predicted by the lower surface energy of CuO_x_ relative to Ag.^[^ ^40^ ^]^

It has been suggested^[^ ^39^ ^]^ that CO_2_ adsorption at the surface of Ag─Cu alloys requires the presence of surface O, which is observed in the low binding energy component at O 1s XPS spectrum of the annealing condition. It cannot be excluded that the C─O and C═O components in C 1s spectra, which are still present after annealing, may participate in this mechanism. The growth of C─O component is followed by the decrease of C═O and adventitious C after dosing CO_2_ and H_2_O (Figure S17, Supporting Information). The interaction of the surface carbon with CO_2_ + H_2_O induces the exchange or conversion of the C─C or C─H species to C─O and/or the volatilization. However, with the laser turned on, the C‐O increase is not followed by a further decrease of adventitious C, indicating a different mechanism. The laser irradiation must be another trigger to further promote the dissociation of CO_2_ at the surface, which is associated with the substitution of Ag for Cu at the surface.

## 4 Probing Morphological Changes

**Figure 3:**
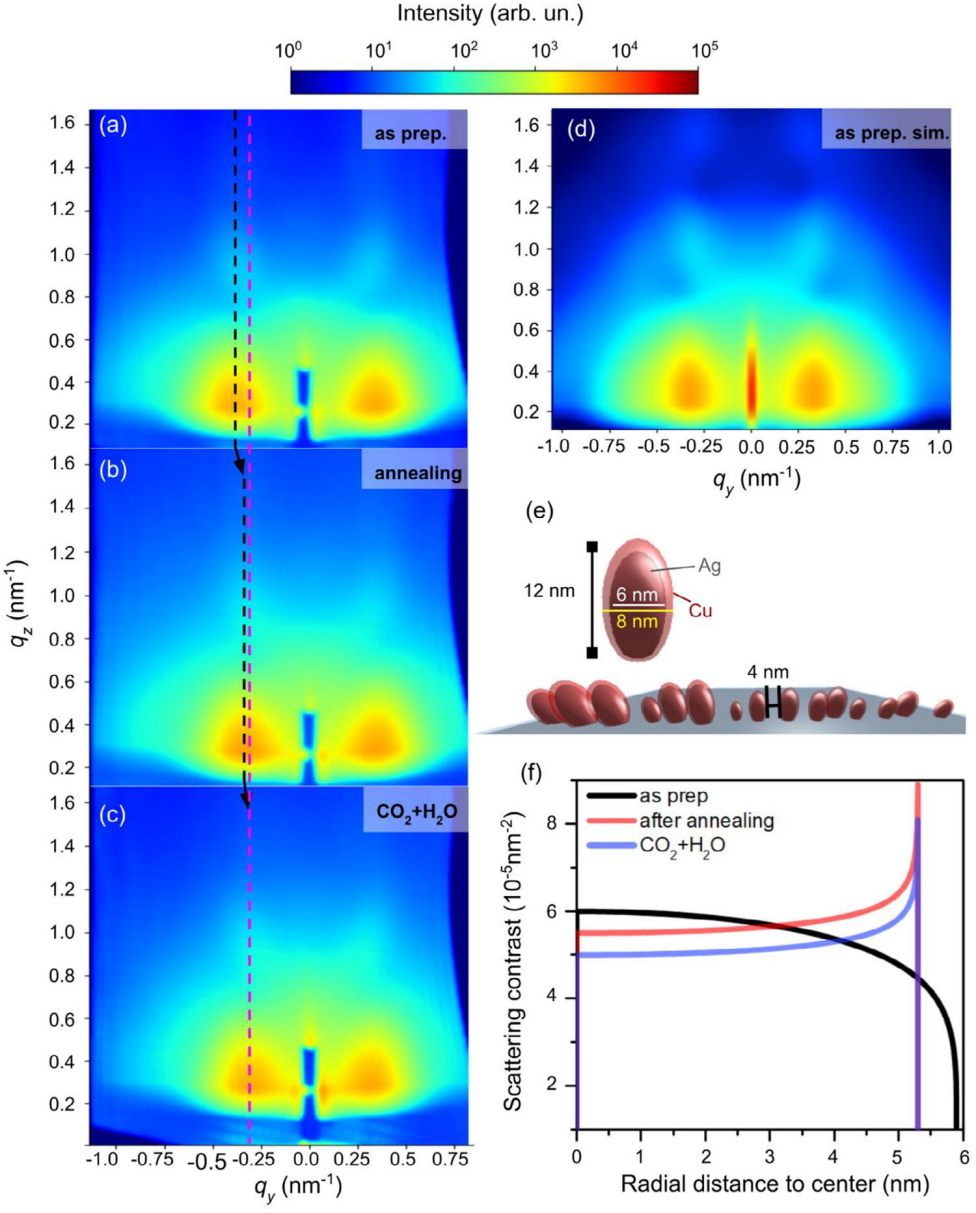
a–c) AP‐GIXS measurements of the sample as prepared, after annealing, and upon 40 mTorr CO_2_ + 40 mTorr H_2_O exposure. Black lines are placed as a guide to the eye centered at the high intensity lobe, while the magenta line shows where linecuts were taken for further analysis. d) Simulated scattering pattern of the as‐prepared sample using BornAgain software, and e) the nanoparticle configuration used for the simulation. f) Scattering contrast as a function of the radial distance to the surface of the average size nanoparticle, obtained after fitting a linecut with the Boucher sphere model.

**Figure 4:**
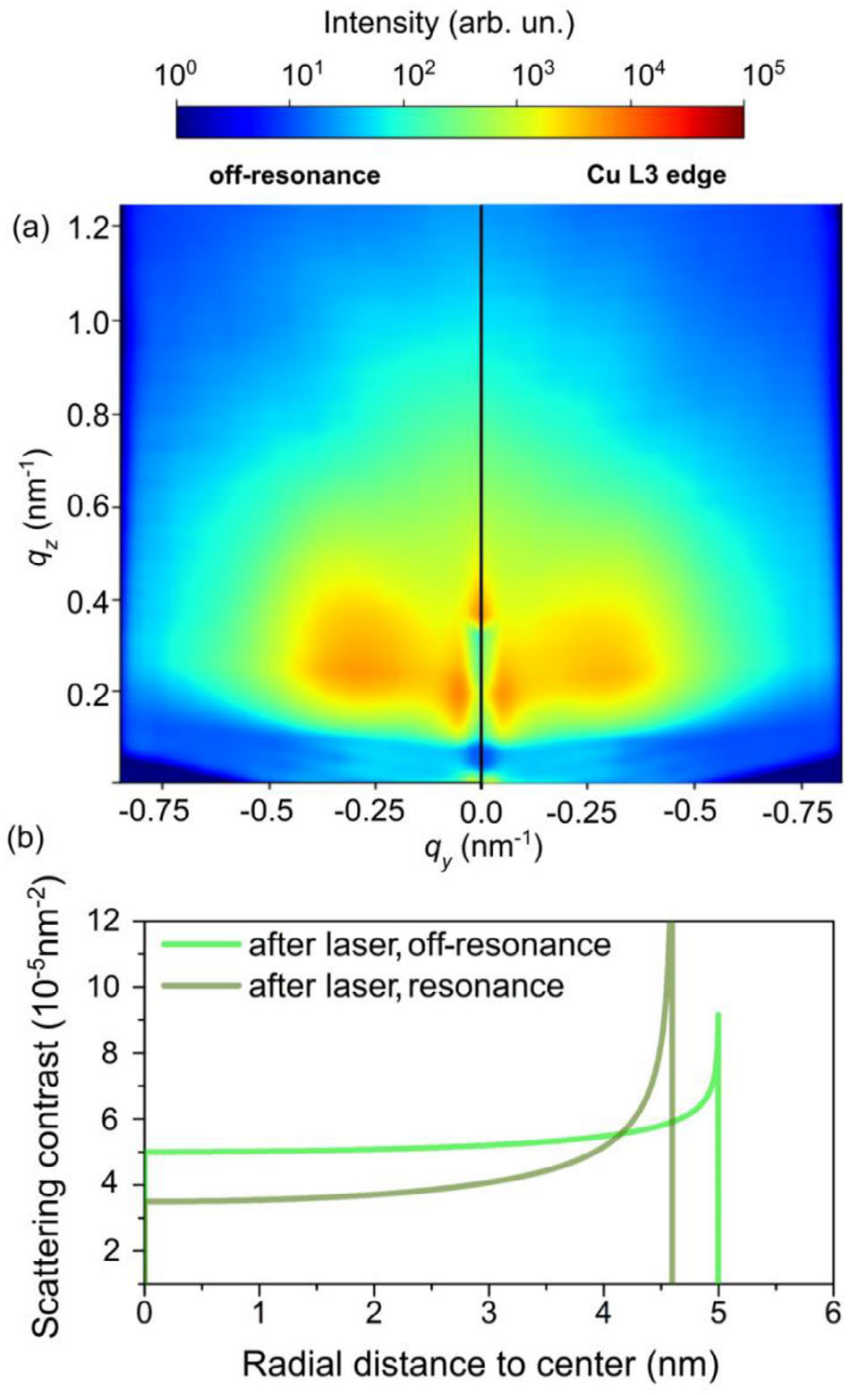
a) Comparison between scattering patterns obtained after 532 nm laser irradiation around Cu L_3_ edge, in off‐resonance and resonance conditions. b) Scattering contrast as a function of the radial distance to the surface of the average size nanoparticle obtained after fitting a linecut with the Boucher sphere model.

AP‐GIXS scattering patterns measured under the same CO_2_RR conditions are shown in Figure 4. The as‐prepared sample (**Figure** **3**a) exhibits broad, intense features around *q_y_* = 0.35 nm^−1^, indicating a strong structure factor affecting the in‐plane scattering components. After annealing (Figure 3b), the structure factor maxima shifts toward the origin, suggesting increased separation between the particles’ center of mass. Moreover, the extension of the out‐of‐plane oscillation above the beamstop footprint indicates a decrease in particle height. Similar trends emerge after CO_2_ + H_2_O exposure (Figure 3c), with further shift of the maxima (Figure S18, Supporting Information) and increased out‐of‐plane oscillation length, suggesting that particles are growing laterally and their elongation along the sample normal is decreasing. This result agrees with TEM measurements of the sample before and after CO_2_RR, as shown in Figure S19 (Supporting Information). Further evidence comes from the Si 2p signal decrease in survey spectra (Figure S20, Supporting Information), supporting the interpretation of scattering data of particles spreading and covering the substrate. We recently demonstrated that monometallic Cu spreads over the support even at relatively low temperatures.^[^ ^41^ ^]^

A simulation of the scattering pattern with BornAgain software package^[^ ^42^ ^]^ (Figure 3d), which shows an excellent agreement with the as‐prepared sample, takes into account a spheroid form factor composed of core‐shell particle geometry, depicted in Figure 3e. The core and shell regions are approximated by the optical constants of Ag(0) and CuO, respectively.^[^ ^43^ ^]^ A recent study showed that the GIXS setup enables obtaining a good estimate for shell thickness.^[^ ^44^ ^]^ The estimated core diameter is of 6 nm, while the shell thickness is of 1 nm, similar to what was previously estimated using AP‐XPS (SESSA simulation) and AFM measurements. The height of the spheroid is ca. 10 nm, with 20% size polydispersity. The as‐prepared sample's interparticle spacing histogram from SEM images shows a maximum in the range of 3–4 nm (Figure S21, Supporting Information). This is considered in the fitting by mixing a paracrystal function with an average distance between the centers of particles of 11 nm.

Linecuts at around *q_y_* = −0.3 nm^−1^ were taken to perform a 1D fitting procedure with the Boucher sphere model (Figure 3f; Figures S22 and S23, Supporting Information). First, the as‐prepared condition shows a scattering contrast that decreases as the radial distance to the surface of the nanoparticle increases, supporting the Ag‐rich core and Cu‐rich shell hypothesis. A linecut taken along *q_y_* = −0.3 nm^−1^ in the simulated BornAgain pattern fitted to the Boucher model (Figure S24, Supporting Information) shows a good agreement with the fitting of the as‐prepared sample (Figure 3f), thus further validating our model. After the annealing procedure, the scattering contrast at the surface is higher than at the core, evidencing that Ag is replacing the Cu atoms at the surface, in full agreement with the AP‐XPS results. After CO_2_ + H_2_O exposure, the scattering contrast has a smaller intensity, which is related to the overall oxidation of the nanoparticles.

Resonant AP‐GIXS captured at the Cu L_3_ edge was measured after laser irradiation in order to estimate the Cu distribution within the nanoparticles (**Figure** **4**a). The pattern at off‐resonance (below the absorption edge) is still heavily influenced by the structure factor contribution. However, at the Cu L_3_ edge white line (934.2 eV), the structure factor weakens as the Cu scattering contribution is suppressed. The smearing of the structure factor is due to a weakening of the hard‐sphere condition, which is embedded into the paracrystal lattice. This happens because the surface is primarily composed of Cu, imposing the limiting condition of particle‐particle separation. After laser irradiation at the off‐resonance condition, the average external radius is shrunk (Figure 4b) as compared to the case before laser irradiation, but the shape is still similar to the previous cases. The fitting of the resonant condition enables identifying that the increase of the scattering contrast at a smaller external shell represents Ag located not fully at the surface anymore, but mostly at the sub‐surface region. The smaller contrast at the core evidences that Cu is also present in that region. Again, it supports the previous observation by AP‐XPS that the laser triggers the replacement of Ag by Cu directly at the surface.

## 5 The Relation between Atomic Diffusion and Morphological Transformation

**Figure 5:**
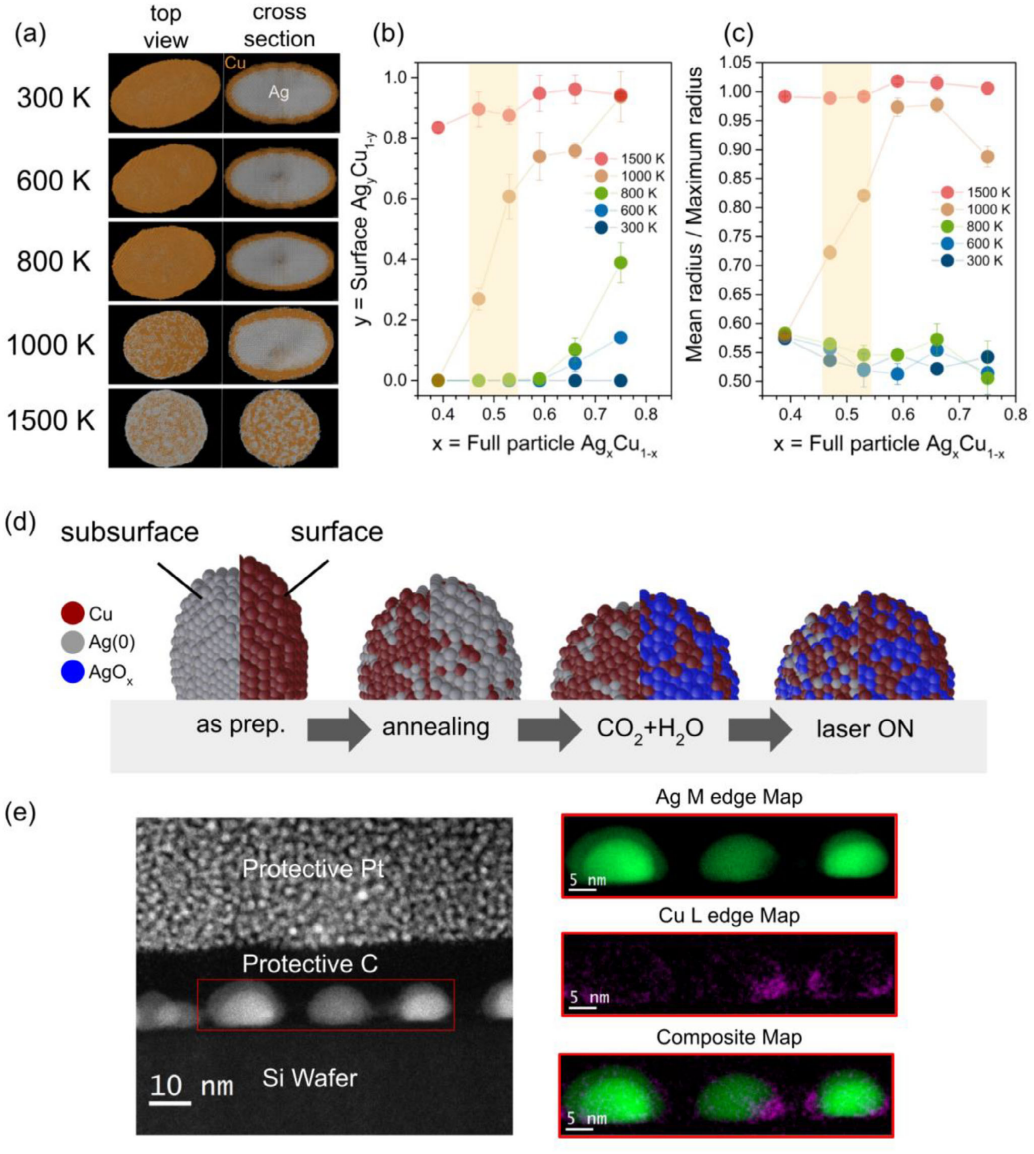
a) Atomic configurations of core‐shell 47% Ag – 53% Cu nanoparticles annealed at different temperatures in MD simulations. b) Surface stoichiometry and c) ratio of mean to maximum particle radius after thermal annealing as a function of the stoichiometry of the full nanoparticle. Shaded areas indicate points closest to experimental observation. d) Schematic representation of the overall transformations observed during CO_2_RR. e) STEM measurements of the Ag‐Cu nanoparticles after CO_2_RR (laser on condition), and respective EELS mapping of the region of interest marked with the red rectangle.

MD simulations of a thermal annealing process were performed on an initially elongated core‐shell nanoparticle with an approximate Ag(0):Cu(0) 1:1 stoichiometry, which is displayed in **Figure** **5**a (see Figure S25, Supporting Information for other compositions). This system is equivalent to the experimental one after annealing in H_2_ atmosphere used to reduce Cu^2+^/Cu^+^ to Cu(0). A general pattern was seen for all nanoparticles regardless of composition, with the Ag atoms replacing the Cu atoms at the surface and the particles becoming more spherical as the temperature is increased (1000–1500 K). Annealing at 1000 K decreases the specific energy of the system (Figure S26, Supporting Information), promoting the most stable atomic configurations during simulation. Because of a significant tendency for Ag segregation, Cu atoms are found in internal pockets even at the highest temperature of 1500 K. Indeed, we recently discovered the formation of internal pockets in bimetallic nanoparticles for a similar system of Ni‐Pd nanoparticles where Pd pockets are formed under H_2_ exposure.^[^ ^45^ ^]^ These temperatures are higher than those used in the experiments (473 K) to enable observing relevant structural transformations within the MD timescale (ps‐ns instead of minutes).

Figure 5b compares the surface Ag‐Cu stoichiometry to the full stoichiometry of a particle, while Figure 5c compares the latter to the mean/maximum radius ratio of a particle. The data suggest that when a particle containing metallic Cu and Ag atoms is subjected to a thermal treatment, there exists a transition temperature (1000 K) at which the mobility of atoms becomes dominant. Diffusion of Cu atoms spontaneously induces changes in morphology. This is further supported by the observation that Ag particles without Cu require annealing at very high temperatures (1500 K) to achieve a significant change in their elongation (Figure S27, Supporting Information). At lower annealing temperatures (300–800 K), all models remain elongated along the main axis and feature a very low concentration of Ag on the surface. After annealing at 1500 K, all particles become nearly spherical, with Ag surface concentration increasing to ≈90%.

An overall representation of the atomic events during CO_2_RR is shown in Figure 5d. The as‐prepared sample is composed of a Cu‐rich shell and an Ag‐rich core. After annealing, the Ag atoms diffuse to the surface region, and the particle begins spreading over the substrate. During CO_2_ + H_2_O exposure, Ag atoms at the surface become oxidized, and Cu atoms diffuse to the surface region to form a Cu‐Ag‐O interface. After the sample is irradiated with the 532 nm laser while being exposed to CO_2_ + H_2_O, the Cu atoms replace Ag atoms at the surface, and the Ag atoms are trapped at the sub‐surface region. The active phase is formed by the Cu overlayer at the Ag‐Cu interface, which is similar to observed in other catalysts such as Pd‐Co.^[^ ^46^ ^]^ STEM‐EELS measurements are shown in Figure 5e and confirm the atomic distribution with the proposed morphology after CO_2_RR. It is interesting to highlight that the structural transformation of Ag‐Cu nanoparticles represents a thermodynamically driven process to minimize energy toward a stable spherical shape in CO_2_RR condition. Furthermore, during the experiments conducted (AP‐XPS with two distinct photon energies and in situ XAS), the surface Cu^+^ active specie was stable during CO_2_RR. Both observations indicate that Ag‐Cu nanoparticles reached a stable configuration, and thus, they should be stable regarding the catalytic activity. Finally, Chang et al. have observed that a reoxidation/reduction of Cu^+^/Cu in Ag‐Cu nanowires by cycling anodic to cathodic potentials induces the atomic diffusion of the species and enhances CH_4_ formation.^[^ ^47^ ^]^ The observation that these light‐induced phenomena promote a similar oxidation step should be leveraged in the design of a photoelectrocatalytic system with improved selectivity and efficiency (see SI for discussion on reaction products).

## 6 Conclusion

Ag‐Cu nanoparticles exposed to photochemical CO_2_RR conditions were studied in situ by multimodal AP‐XPS/ AP‐GIXS measurements, revealing details about the chemical and morphological reconstructions on atomic level. Cu and Ag atoms reorganize within the nanoparticles while the overall shape of nanoparticles reaches equilibrium in the form of hemispheres, which was corroborated by MD simulations. Changes of nanoparticles’ overall morphology are accompanied by the formation of a Cu‐Ag‐O interfacial phase during CO_2_ + H_2_O exposure. Moreover, visible light irradiation further promotes oxidation of Cu atoms, which partially replace Ag at the surface, while Ag is trapped in the sub‐surface layers. We identified these processes as responsible for enhanced activity and selectivity of the Ag‐Cu system. This photochemically induced transformation leads then to charge transfer between adsorbents and nanoparticles, but other factors, like the heat generated during the irradiation process, also need to be considered.

To disentangle different contributions, future studies will include red‐ and blue‐shifting the irradiating light. It will also be interesting to explore whether similar bimetallic systems, such as Cu‐Au and Cu‐Pt, exhibit comparable behavior. Furthermore, any kinetic effects related to the diffusion of Cu and Ag atoms are evidently hidden during these experiments, since the measurements are not time‐resolved. Atomic diffusion is closely linked to morphology kinetics, and thus measuring time‐resolved AP‐GIXS and/or AP‐XPS would provide data related to the rate of diffusion.

Our findings and newly applied methodology of 2 co‐located simultaneous probes can aid in the design of catalysts suited for cathodic electrochemical currents. Since Cu(0) is the stable phase during typical electrochemical CO_2_RR, triggering oxidation via light exposure could enhance catalyst stability. Our results show that such a catalytic system can be dynamically controlled under light‐off/light‐on conditions to harness the diffusion mechanism and therefore stabilize the desired intermediate states.

## 7 Experimental Section

The Ag‐Cu nanoparticles were prepared on Si(111) p‐type doped wafers using a commercial thermal evaporator system, Denton DV‐502A at the Molecular Foundry, Lawrence Berkeley National Laboratory (LBNL). The wafers were sonicated with isopropanol and water, followed by drying in N_2_ gas flow. The evaporator system was evacuated using a scroll pump and a turbomolecular pump, reaching a stable pressure typically ≈10^−5 ^Torr after 2 h. Ag was first evaporated until a thickness of 2 nm was reached with a rate of 0.5 Å s^−1^, measured using a quartz crystal microbalance. The sample plate was then heated for 2 h at 60 °C to outgas and then to 250 °C during 12 h to form Ag nanoparticles. Afterward, the same procedure was used to evaporate 0.5 nm thickness of Cu, which was annealed at 250 °C for another 12 h.

AP‐XPS measurements were taken at beamline 9.3.2 at ALS,^[^ ^48^ ^]^ which is equipped with a VG‐Scienta R4000 HiPP electron analyzer. All the measurements were performed using a 695 eV photon beam energy. Analyzer pass energy of 100 eV was used with step sizes of 0.1 and 0.5 eV for the high‐resolution and survey spectra, respectively. The AP‐XPS spectra were collected in the survey, Cu 3p, Ag 4p, Ag 3d, Si 2p, C1s, O1s, and valence band electronic regions. The sample was initially measured in the as‐prepared condition, which was followed by dosing 20 mTorr H_2_ and annealing to 200 °C. At this temperature, the sample remained 30 min, and a second set of AP‐XPS measurements was taken. After cooling down to RT in UHV, another set of measurements was taken. Then, 40 mTorr CO_2_ + 40 mTorr H_2_O were dosed into the main chamber for 30 min. After measuring the sample in this condition, a 532 nm DPSS laser was coupled to a power supply operating at 3.0 V and 0.3 A. The laser irradiated through the nozzle of the analyzer over the sample. A temperature rise of 5 °C on the sample was measured after 5 min of laser irradiation with the aid of a thermocouple, stabilizing at this temperature during laser operation. This condition was kept for 30 min, and new AP‐XPS measurements were conducted after this period with the sample exposed to CO_2_ + H_2_O and the laser on.

AP‐XPS, AP‐GIXS, and in situ XAS measurements were performed at APPEXS endstation at 11.0.2 beamline at ALS.^[^ ^49^, ^50^ ^]^ The APPEXS endstation is equipped with a SPECS PHOIBOS 150 NAP electron analyzer and an Andor iKon‐L CCD mounted on the biaxial quasi‐spherical manipulator to collect the scattered X‐rays. A 532 nm laser probe (Spectra Solutions, Inc.) was placed inside the main chamber and operated at 800 mW. The AP‐XPS and AP‐GIXS measurements were taken using a 1240 eV photon energy, corresponding to an X‐ray wavelength of 1 nm. The measurements were performed in grazing incidence using a 1° angle relative to the surface, below the critical angle of Si substrate (≈1.2°). AP‐XPS measurements were collected in the survey, Cu 3p, Ag 4p, Ag 3d, Si 2p, C1s, O1s, and valence band electronic regions. A step of 1 eV and 0.1 eV, and pass energy of 10 eV were used for the survey and high‐resolution spectra, respectively. For AP‐GIXS, the scattered X‐rays are collected at ±12° along in‐plane and 24° out‐of‐plane directions. In situ XAS measurements were performed at Cu L edge, from 920 eV to 960 eV, in total electron yield/drain‐current mode using thermocouple wires connected to the Si wafer, electrically isolated from the sample holder. The signal was obtained using a pico‐Amperimeter configured with a 20 nA V^−1^ sensitivity, with incremental steps of 0.15 eV and a collection time of 0.5 s per point. Two spectra were averaged for each different condition. The sequence of experimental conditions applied to the samples in APPEXS setup was the same as during AP‐XPS measurements performed at beamline 9.3.2. A similar temperature rise was observed when the laser was turned on.

SEM images were obtained at Molecular Foundry‐LBNL using a Zeiss Gemini Ultra‐55 microscope. The samples were measured before and after the AP‐XPS and AP‐GIXS measurements. The SEM images were obtained by detecting secondary electrons. The images were analysed with ImageJ software by selecting an appropriate threshold and automatic detection of grains.^[^ ^51^ ^]^ AFM measurements were conducted at the Imaging Facility of the Molecular Foundry‐LBL using a Asylum Jupyter AFM, and a PEAKFORCE‐HIRS‐F‐A tip from BrukerNano. This high‐resolution probe features a nominal tip radius of 1 nm. The achievable spatial resolution in AFM is fundamentally linked to the sharpness of the probe tip. The AFM scans were performed with a lateral pixel size of 2 nm. The AFM was operated in tapping mode, with a drive amplitude of 2 mW, 100 mV setpoint voltage, scan rate of 0.75 Hz, and 256 × 256 resolution. The optical properties of the samples were studied using ultraviolet–visible (UV–Vis) total reflectance spectra at CEOMAT‐UFRGS on a UV—vis–NIR spectrophotometer Cary 5000 (Agilent) in the wavelength range of 300–800 nm using an integrating sphere (DRA – 1800) with a sphere diameter of 150 mm. Measurements were taken of the sample grown on top of the Si substrate before and after exposure to every condition at beamline 11.0.2, and of the bare, clean Si substrate.

Due to the presence of Cu in the sample Mo FIB Lift‐out TEM grids purchased from Ted Pella were used in the electron microscopy characterization. To avoid contamination and damage to the samples, the empty Mo FIB grids were cleaned using a 4 min plasma cleaning for the TEM lamella preparation. Thin electron transparent lamellas of Cu–Ag nanoparticles on Si were prepared using an FEI Helios G4 UX dual‐beam Focused Ion Beam (FIB) instrument at the National Center for Electron Microscopy (NCEM). Samples were examined both in their as‐prepared state and after CO_2_RR (laser on). To minimize surface damage, a 100 nm carbon (C) protective layer was first deposited, followed by a 2 µm platinum (Pt) layer. The C layer also enhanced nanoparticle visualization. Lamellas were welded onto molybdenum FIB Lift‐Out TEM grids and initially thinned from 2 µm to <100 nm for final characterization. Thinning was performed via multiple gallium‐ion beam thinning steps, using progressively lower accelerating voltages and beam currents to reduce ion‐induced damage. The accelerating voltage was decreased from 30 kV to 1 kV over 4–5 thinning steps, while the corresponding beam current was reduced from 0.75 nA to 44 pA.

The electron energy loss measurements for compositional mapping were performed using the TEAM 1 microscope (double‐aberration‐corrected Thermo Fisher Scientific Titan 80–300) at NCEM. The EELS measurements were acquired using a Gatan Continuum spectrometer at 300 kV, with a convergence angle of ≈17.1 mrad and a collection angle of 82 mrad. The width of the zero‐loss peak was measured to be 1.5 eV, and a dispersion of 0.35 eV per channel was used to collect the spectra. To prevent contamination and damage, multiple short dwell time (≈1.7 ms) EELS passes were collected and summed up after drift correction EELS. The compositional maps were analyzed and generated using Gatan DigitalMicrograph software v3.6.

KOLXPD software was used for the AP‐XPS data analysis. For the fitting of the high‐resolution spectra, a Shirley‐type background was subtracted. Voigt functions were used, where the Gaussian width of the peaks was fixed at the same value for each electronic region and the same excitation energy. The Lorentzian width was fixed for the same chemical component in a given electronic region for all the measured conditions. The relative binding energy of each chemical component was also fixed during the procedure. AP‐GIXS raw data treatment is described in the Supporting Information. BornAgain software was used to simulate the AP‐GIXS scattering patterns. A spherical detector was considered, with a 1° grazing‐incidence X‐ray beam on the sample. Different in‐plane and out‐plane linecuts were taken in the measured data to compare with the simulated pattern. SASfit software was used to perform the fitting procedure of selected measured data linecuts. The fitting used a fixed constant background and a Boucher sphere form factor. Considering the Boucher sphere model present in the SASfit package,^[^ ^52^ ^]^ which is based on a study of alloy particles.^[^ ^53^ ^]^ The fitting takes into account polydispersity by considering a log‐normal size distribution and a variable scattering contrast along the radius of the nanoparticles based on a single parameter (fitted data are shown in Figures S22 and S23, Supporting Information). The scattering contrast as a function of the radial distance from the center to the surface of the nanoparticle (with radius equal to the average size of the particle distribution) obtained after the fitting is shown in Figure 3f.

Classical molecular dynamics (MD) simulations were carried out using the LAMMPS package^[^ ^54^ ^]^ to study the atomic structure of AgCu nanoparticles of varying diameters and compositions annealed at different temperatures. The interatomic interactions were described by the EAM (Embedded‐Atom Method) potential,^[^ ^55^ ^]^ with a suitable parameterization for Ag and Cu,^[^ ^56^ ^]^ which describes well the structure of individual phases and their alloys. To further elucidate the effect of annealing temperature and atomic composition on the features of Ag‐Cu nanoparticle surfaces, the study created core‐shell (Ag‐Cu) ellipsoidal nanoparticles with characteristic dimensions of 12 ± 1 × 7 ± 1 × 7 ± 1 nm^3^ (dimensions slightly vary, depending on the composition), with compositions of 25, 34, 41, 47, 53, and 61% Cu (keeping the total characteristic dimensions and varying the shell thickness, the number of atoms varies from 17 400 to 33 700). The initial systems were submitted to thermal annealing at different temperatures (300, 600, 800, 1000, and 1500 K) starting from 0.1 K, using heating and cooling stages of 500 ps and equilibration steps of 3 ns (with timesteps of 5–8 fs). Higher temperatures than those used in experiments are employed to accelerate structural transformations and observe relevant phenomena in the timescale accessed by MD (nanoseconds, instead of minutes in the real experiments). Therefore, their absolute values should not be taken for direct comparison purposes, and only the trends must be considered (i.e., analyze which transformations occur or do not occur as the annealing temperature is increased). The temperature was controlled using the Nosé‐Hoover thermostat. The system energy was monitored to ensure that the annealing time was long enough to promote a steady value. The final morphology, characteristic dimensions, and surface composition of the nanoparticles were analyzed after annealing. Further information is given in the Supporting Information.

## Conflict of Interest

The authors declare no conflict of interest.

## Supporting information



Supporting Information

## Data Availability

The data that support the findings of this study are available from the corresponding author upon reasonable request.
