# Supplementary material for: Shapeshifting Nanocatalyst for CO2 Conversion
Source: Adv Mater. 2025 Sep 18;38(2):e09814. doi: 10.1002/adma.202509814 (PMC12783975; doi:10.1002/adma.202509814)
Supplement: Supplementary file 1 — Supporting Information [file ADMA-38-e09814-s001.pdf]

ADVANCED MATERIALS

Supporting Information

for *Adv. Mater.*, DOI 10.1002/adma.202509814

Shapeshifting Nanocatalyst for CO_2 Conversion

Gustavo Zottis Girotto, Maximilian Jaugstetter, Dongwoo Kim, Lívia P. Matte, Tara P. Mishra, Mary Scott, Ruan M. Martins, André R. Muniz, Miquel Salmeron, Slavomir Nemsak and Fabiano Bernardi**

Supporting Information

Shapeshifting Nanocatalyst for CO_2 Conversion

Gustavo Zottis Girotto,[1,2] Maximilian Jaugstetter,[3] Dongwoo Kim,[2,4] Lívia P. Matte,[3] Tara P. Mishra,[3] Mary Scott,[3] Ruan M. Martins,[5] André R. Muniz,[5] Miquel Salmeron,[3] Slavomir Nemsak,[2,6,*] Fabiano Bernardi[1,*]

[1] Programa de Pós-Graduação em Física, Instituto de Física, Universidade Federal do Rio Grande do Sul, Porto Alegre, RS, Brazil

[2] Advanced Light Source, Lawrence Berkeley National Laboratory, Berkeley, CA, USA

[3] Materials Science Division, Lawrence Berkeley National Laboratory, Berkeley, CA, USA

[4] Department of Physics and Photon Science, Gwangju Institute for Science and Technology, Gwangju, South Korea

[5] Department of Chemical Engineering, Universidade Federal do Rio Grande do Sul, Porto Alegre, RS, Brazil

[6] Department of Physics and Astronomy, University of California, Davis, CA, USA

Gwyddion software[1] was used for analysis of AFM images. Grains were selected using the automatic edge detection function. Figure S1(a) shows the top view of the typical image shown in Figure 1(a) of the main text. Figure S1(b) shows the grain selected for further analysis. Figure S1(c) shows the height of each individual grain as a function of its equivalent radius.

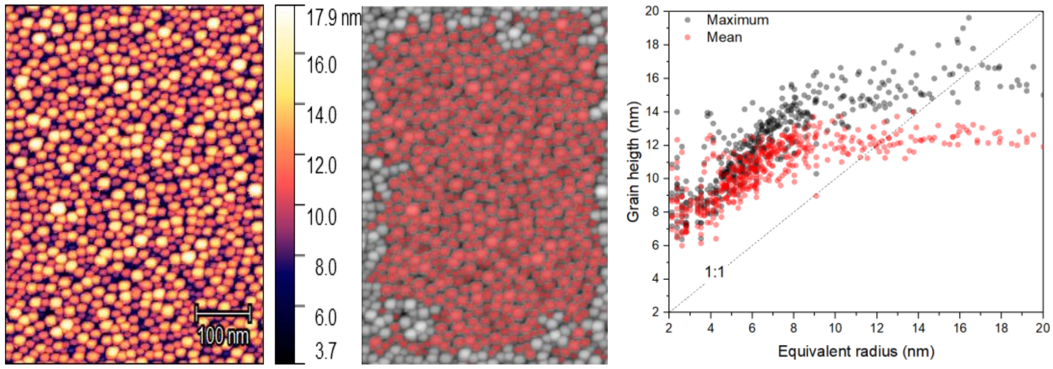

Figure S1: Analysis of a typical AFM image of the sample after exposure to CO_2RR at 11.0.2 beamline. (a) top view, (b) grain selection, (c) height as a function of equivalent radius of each grain.

For the artificial photosynthesis reaction, Ag-Cu/Si nanoparticles were inserted in an in-house reactor[2], exposed to 1 atm H_2 atmosphere and annealed to 200 ºC for 1 h. After this, the sample was cooled to room temperature and the H_2 atmosphere was replaced by 1 atm CO_2 atmosphere. Then, 10 mL of ultrapure liquid water was added to the reactor while CO_2 was continuously bubbled. A lamp of 10 W power and λ = 365 nm combined with another of 50 W and λ = 532 nm (both at 5 cm distance) were used as the photon sources for the reaction during 5 h at room temperature.

Fourier Transform Infrared Spectroscopy (FTIR) measurements were performed in the centrifuged liquid after reaction at the Laboratório de Materiais Poliméricos (LAPOL-UFRGS) using the Spectrum 1000 Perkin Elmer equipment. The FTIR measurements were performed with 4 cm^{-1} resolution and the result is from the average of 32 scans, subtracted from a measurement of blank H_2O solution.

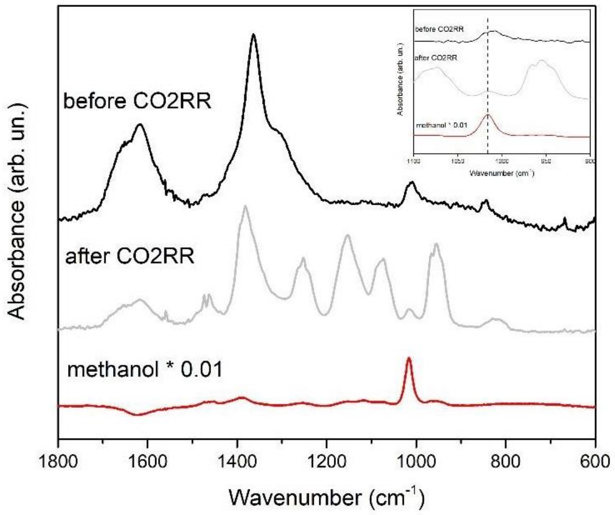

Figure S2 – FTIR spectroscopy measurements of the liquid used in the CO_2RR for Ag-Cu/Si nanoparticles.

GC-FID measurements were conducted using a Shimadzu GC-2010 Plus equipment with an Agilent J&W DB-WAXETER column (30 m x 0.25 mm x 0.25 µm). 1.50 mL/min N_2 was used as carrier gas.

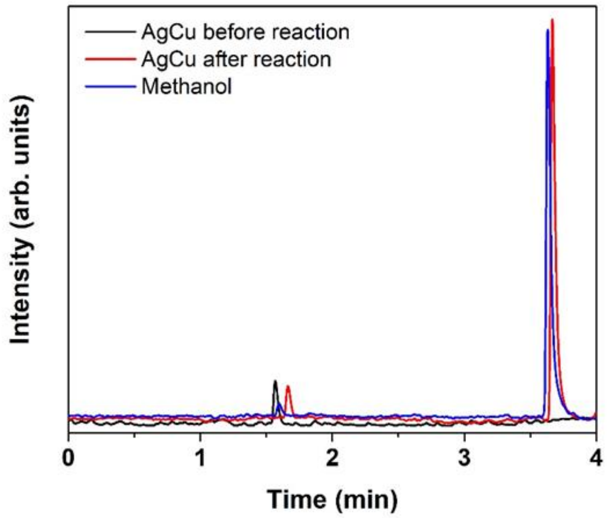

Figure S3 – GC-FID chromatogram of the solution of the CO_2RR before and after reaction of Ag-Cu/Si nanoparticles and a methanol standard for comparison purposes.

For comparison purposes, Ag-Cu nanoparticles were also synthesized through a wet synthesis method (chemical route). In a typical synthesis, 100 mg of glucose dispersed in 10 mL of ultrapure water is mixed with a 30 mL 0.03 M NaOH solution. After stirring the solution for 5 min at 500 rpm, 30 mg of $Cu(NO_3)_2$ dispersed in 2 mL of ultrapure water is added. Then, after 2 min, 30 mg of $AgNO_3$ dispersed in 10 mL of ultrapure water is added. The solution is stirred for 5 min, and then heated from 20 ºC to 60 ºC with a 1.5 ºC/min rate. After 10 min in 60 ºC, the solution is left to cool to room temperature. The sample is washed and centrifuged at 4000 rpm for 10 min by mixing ethyl acetate, and centrifuged again using only ultrapure water. Finally, the sample is dried under vacuum for 12 h. After drying, a hard pellet is formed, which is then ground using pestle and mortar. This synthesis gives typically around 40 mg of powder.

Figure S4(a) shows TEM images of the Ag-Cu nanoparticles synthesized via chemical route. The TEM images were obtained at CM-UFMG in a Tecnai G2-12 microscope working at 120 kV. The powder was dispersed in water, and a drop of the solution was placed over a carbon coated Cu grid. The nanoparticle sizes were estimated using the average Feret diameter, which was manually measured in ImageJ software (2019 version). The size distribution obtained is shown in Figure S4(b). The standard deviation obtained indicates that the distribution is very polydisperse. This reduces the information depth that analysis of X-ray scattering experiments is able to extract. Therefore, the study of a model system with GIXS allows understanding of the overall picture. Figure S4(c) shows a high-resolution TEM

image of one typical nanoparticle, and Figure S4(d) shows the FFT of the indicated regions that comes from Ag(0) at the core and Cu_2O at the shell region.

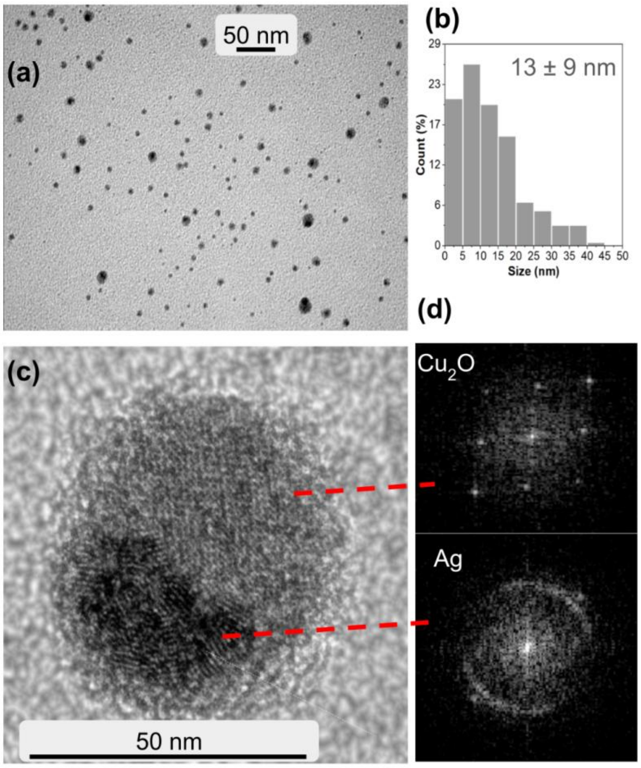

Figure S4: (a) Typical TEM images of the AgCu nanoparticles synthesized via chemical route and (b) the corresponding size distribution of the nanoparticles. (c) HRTEM image of a Ag-Cu nanoparticle with, (d) the corresponding FFT of the indicated regions.

The AP-XPS measurements were conducted at beamline 9.3.2 of ALS using the same conditions for the thermal evaporated sample. The powder was dissolved in ultrapure water, and centrifuged at 1000 rpm for 1 min to remove bigger grains. Then the supernatent containing nanoparticles is placed in a small vial, and a Si wafer was placed facing downwards touching the liquid interface. After 1 h, the wafer was removed and immediately dried with N_2 gas flow. Figure S5 shows SEM image of the nanoparticles dispersed over the Si wafer.

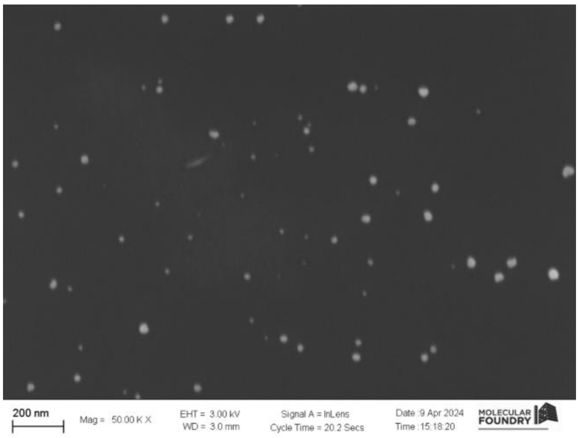

Figure S5: SEM image of the Ag-Cu nanoparticles synthesized via chemical route supported on Si wafer.

The AP-XPS measurements at the Cu 3p and Ag electronic regions of the Ag-Cu nanoparticles synthesized via the chemical route (Figure S4) over the Si substrate with 695 eV photon energy are shown in Figure S6. Because the nanoparticles present a thicker Cu shell, the total intensity of the Ag 4p region is very small, and thus the comparison was made directly to the Ag 3d regions. Similar treatments were applied to what was measured with the thermally evaporated samples and the behavior is quite similar to that observed for the thermal evaporated sample. Firstly, the as prepared sample shows Cu^{2+} and Cu^{+}/Cu(0) chemical components. The Cu^{2+} components disappears during annealing in H_2 atmosphere. The Cu oxidation state doesn't change during the subsequent conditions. Figure S7 shows the Cu/Ag ratio calculated for the different conditions. The normalized Cu/Ag ratio drops after annealing and it rises during exposure to CO_2 + H_2O, as observed for the thermal-evaporated sample.

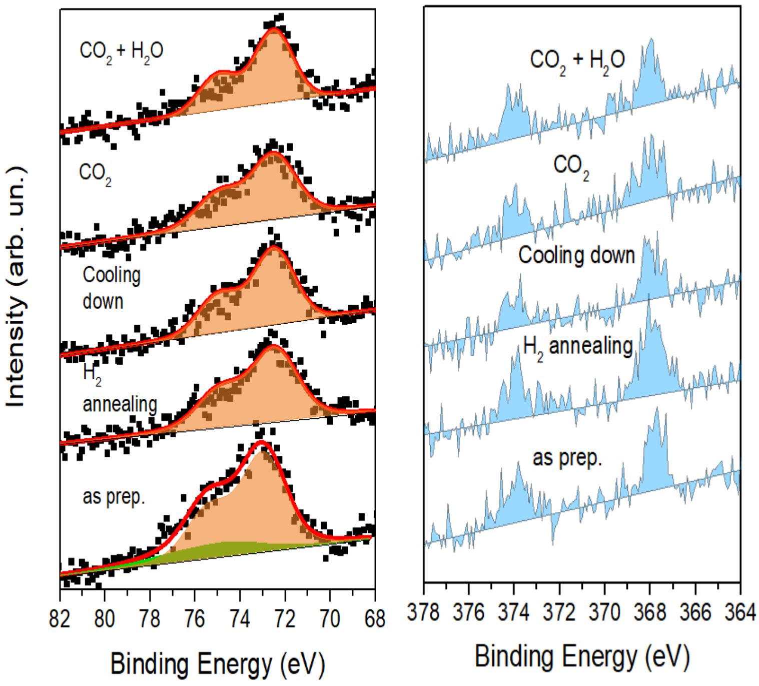

Figure S6: AP-XPS measurements at (a) Cu 3p, and (b) Ag 3d electronic regions for the Ag-Cu nanoparticles synthesized via chemical route.

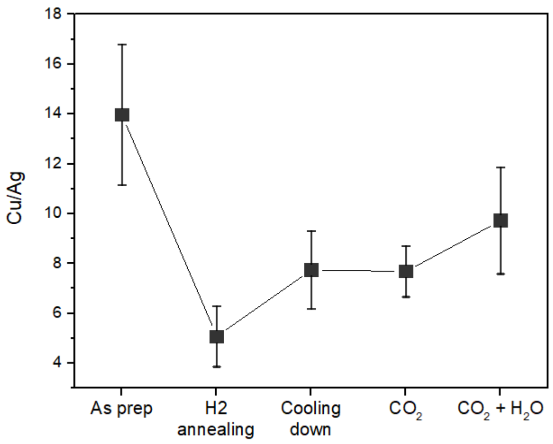

Figure S7: Normalized Cu/Ag ratio obtained from AP-XPS measurements of the Ag-Cu nanoparticles synthesized via chemical route.

For the artificial photosynthesis reaction, 100 mg of 50 wt% of Ag-Cu/TiO_2 nanoparticles were dispersed on 10 mL of water with 0.1 M $NaHCO_3$ solution. The solution kept in an in-house reactor that contains a gas headspace and a quartz window. CO_2 was bubbled for 10 min through the solution. A lamp of 10 W power and λ = 365 nm combined with another of 50 W and λ = 532 nm (both at 5 cm distance) were used as the photon sources for the reaction during 5 h at room temperature.

Figure S8 shows the FTIR measurements where it is observed the appearance of a band that matches the one present in a 10%v/v in H_2O methanol solution alongside carbonate related bands (from $NaHCO_3$), showing that methanol is clearly produced from this reaction.

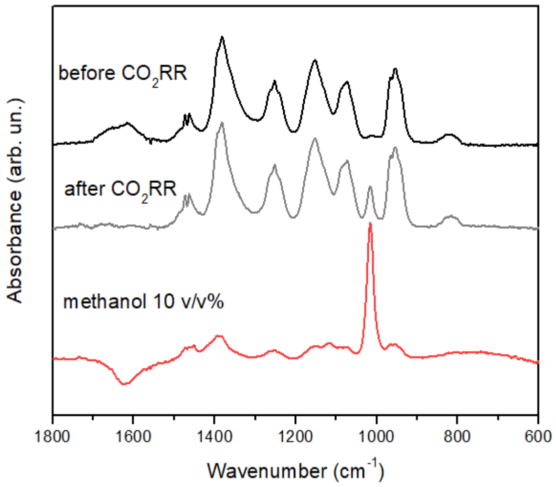

Figure S8: FTIR spectroscopy measurements of the liquid used in the CO_2RR of Ag-Cu/TiO_2 nanoparticles synthesized via chemical route.

Figure S9 shows the normalized Cu/Ag ratio of the Ag-Cu/Si nanoparticles synthesized through the thermal evaporation method and obtained after fitting the Cu 3p and Ag 4p AP-XPS regions from the measurements at beamline 11.0.2. Again, the annealing procedure allows the diffusion of Ag atoms to the surface, decreasing the Cu/Ag ratio in comparison to the as prepared case. After dosing CO_2 + H_2O, there is a slight increase in the ratio, agreeing with what was observed previously. There is also an important increase of this ratio in the laser on condition. This again shows that laser irradiation triggers Ag substitution by Cu at the surface.

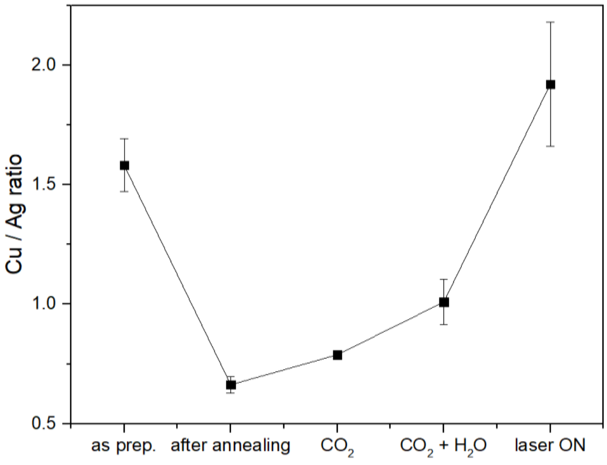

Figure S9: Normalized Cu/Ag ratio from Cu 3p and Ag 4p AP-XPS electronic regions as a function of the treatment employed..

Figure S10 shows the result of a simulation obtained by using SESSA software to calculate the Cu/Ag ratio of different Ag-Cu core-shell structures. The simulation takes into account the general geometrical parameters in 9.3.2 beamline. The areas obtained by the simulation were fitted using KOLXPD software using the same procedure applied to the experimental data.

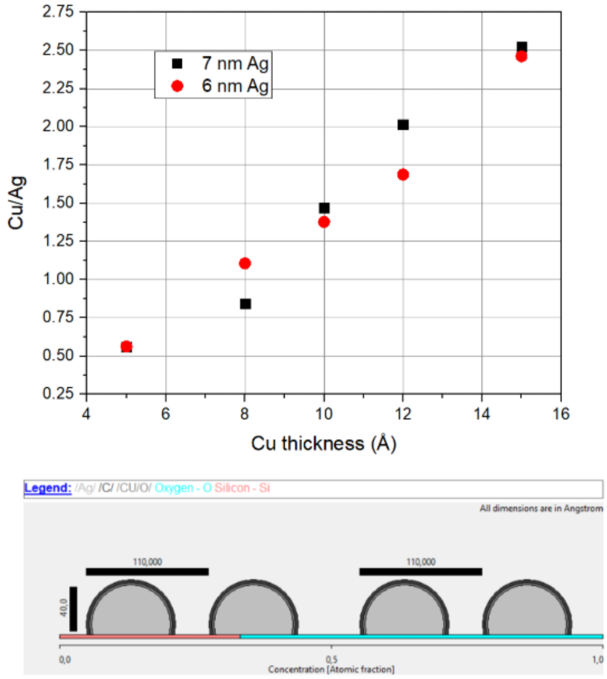

Figure S10: Simulation of Cu/Ag ratio obtained through SESSA as a function of Cu shell thickness with an Ag core of around 6 or 7 nm diameter.

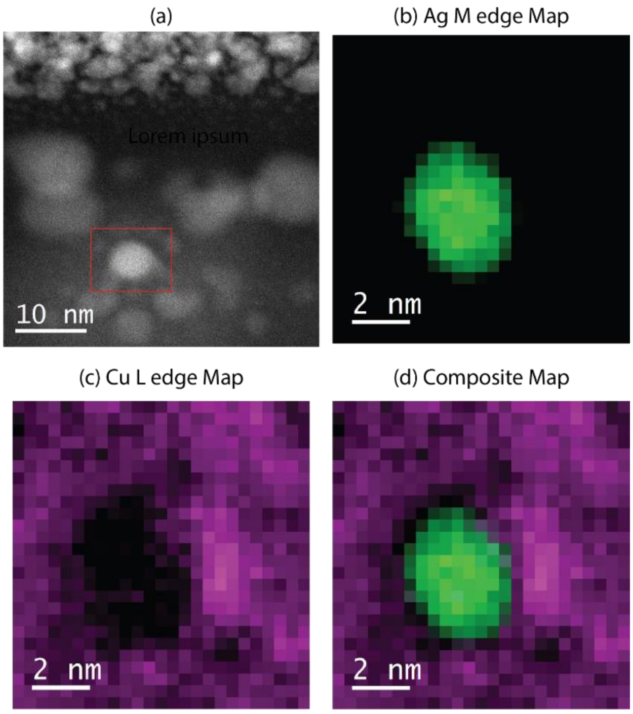

Figure S11: STEM-EELS measurements of the as-prepared Ag-Cu/Si nanoparticles. (a) HAADF image of the nanoparticles, (b) Ag M edge signal map, (c) Cu L edge signal map and (d) superimposition of the Ag and Cu signal obtained from the EELS mapping.

Table S1. Binding energy position, fraction and FWHM of each chemical component used in the fitting of the Cu 3p + Ag 4p AP-XPS regions.

	Binding Energy (eV)			Fraction			FWHM (eV)		
	Ag 4p	Cu^+	Cu^{2+}	Ag 4p	Cu^+	Cu^{2+}	Ag 4p	Cu^+	Cu^{2+}
As prepared	57.7	75.2	76.7	0.25	0.25	0.50	8.0	1.9	3.2
After annealing	57.4	74.7	76.2	0.39	0.58	0.03	8.0	1.9	3.2
CO_2	57.4	74.6	76.1	0.39	0.55	0.06	8.0	1.9	3.2
$CO_2 + H_2O$	57.3	74.4	75.9	0.38	0.54	0.07	8.0	1.9	3.2

laser on	57.5	74.3	75.8	0.36	0.54	0.10	8.0	1.9	3.2

Table S2. Binding energy position, fraction and FWHM of each chemical component used in the fitting of the Ag 3d AP-XPS region.

	Binding Energy (eV)		Fraction		FWHM (eV)	
	AgO_x	Ag(0)	AgO_x	Ag(0)	AgO_x	Ag(0)
As prepared		368.2		1.00		1.0
After annealing		367.9		1.00		1.0
CO_2	367.5	368.1	0.26	0.74	1.1	1.0
$CO_2 + H_2O$	367.4	368.0	0.54	0.46	1.1	1.0
laser on	367.3	367.9	0.54	0.46	1.1	1.0

Table S3. Binding energy position, fraction and FWHM of each chemical component used in the fitting of the C 1s AP-XPS region.

	Binding Energy (eV)					Fraction					FWHM (eV)				
	C-C	C-O	C=O	CO_2	CO_3	C-C	C-O	C=O	CO_2	CO_3	C-C	C-O	C=O	CO_2	CO_3
As prepared	285.3	286.0	288.4		289.9	0.45	0.17	0.06		0.31	1.5	1.7	3.3		1.69
After annealing	285.0	285.7	288.1			0.52	0.27	0.21			1.5	1.7	3.3		
CO_2	284.9	285.6	288.0	294.0		0.38	0.36	0.20	0.06		1.5	1.7	3.3	1.5	

$CO_2 + H_2O$	284.7	285.4	287.8	294.0	0.23	0.54	0.19	0.04	1.5	1.7	3.3	1.5
laser on	284.7	285.4	287.8	294.0	0.22	0.56	0.18	0.04	1.5	1.7	3.3	1.5

Table S4. Binding energy position, fraction and FWHM of each chemical component used in the fitting of the O 1s AP-XPS region.

	Binding Energy (eV)					Fraction					FWHM (eV)				
	CuO_x	Ag-Cu-O	SiO_x	H_2O	CO_2	CuO_x	Ag-Cu-O	SiO_x	H_2O	CO_2	CuO_x	Ag-Cu-O	SiO_x	H_2O	CO_2
As prepared	529.8		531.7			0.12		0.88			1.6		1.5		
After annealing	530.5		532.5			0.07		0.93			1.6		1.5		
CO_2	530.6	532.1	532.6		536.5	0.05	0.36	0.54		0.04	1.6	1.8	1.5		1.5
$CO_2 + H_2O$	530.7	532.2	532.6	535.5	536.6	0.04	0.59	0.33	0.01	0.03	1.6	1.8	1.5	1.5	1.5
laser on	530.6	532.1	532.5	535.4	536.5	0.09	0.54	0.33	0.01	0.03	1.6	1.8	1.5	1.5	1.5

The thermal evaporated Ag-Cu/Si nanoparticles were inserted in an in-house reactor[2], exposed to 1 atm H_2 atmosphere and annealed to 200 ºC for 1 h. After this, the sample was cooled to room temperature and the H_2 atmosphere was replaced by 1 atm CO_2 atmosphere. Then, 10 mL of ultrapure liquid water was added to the reactor while CO_2 was continuously bubbled. A 532 nm DPSS laser was turned on and irradiated the Ag-Cu/Si surface for 30 min. After this, the sample was placed inside the load lock of the UHV XPS chamber while still wet and left to dry in low vacuum. The XPS measurements were conducted using an Omicron SPHERA analyzer equipped with an Al Kα X-ray source (hν = 1486.7 eV). The base pressure during the measurements was maintained at 10^{-8} mbar. A pass energy of 10 eV and energy step of 0.1 eV with a dwell time of 0.2 s were used.

Figure S12 shows the Cu $2p_{3/2}$ and Ag 3d XPS electronic regions of the Cu-Ag/Si nanoparticles in the as-prepared and after reaction conditions. The Cu $2p_{3/2}$ XPS region shows the presence of Cu^+/Cu(0) and Cu^{2+} components in the as prepared case. After reaction, the major component is Cu^+/Cu(0), in agreement with AP-XPS data shown in Figure 2. In this case, photoelectrons coming from the Cu $2p_{3/2}$ region present a kinetic energy of around 550 eV, which is almost the same kinetic energy of photoelectron from Cu 3p in Figure 2 (610 eV). It gives an inelastic mean free path (λ) of around 10 Å in both cases, so almost the same region is probed in both measurements. On the other hand, Ag 3d XPS region of Figure S12 shows only the presence of Ag(0) component before and after reaction, while Figure 2 shows the appearance of AgO_x after reaction. However, the probed depth in the measurement of Figure S12 is λ = 15 Å, while in Figure 2 it is much more surface sensitive with λ = 8 Å. Indeed, AP-XPS measurements with hν = 1240 eV and λ = 11 Å, shown in Figure S14, already showed a relative decrease of the AgO_x component for larger λ values. It occurs because the AgO_x component becomes insignificant as compared to the Ag(0) one, so it is barely seen in this condition.

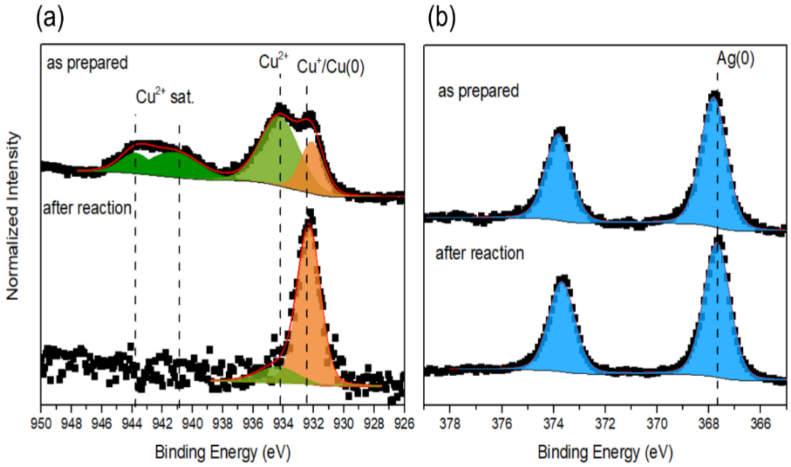

Figure S12 – (a) Cu $2p_{3/2}$ and (b) Ag 3d quasi in-situ XPS measurements of the Ag-Cu/Si nanoparticles before and after realistic CO_2RR.

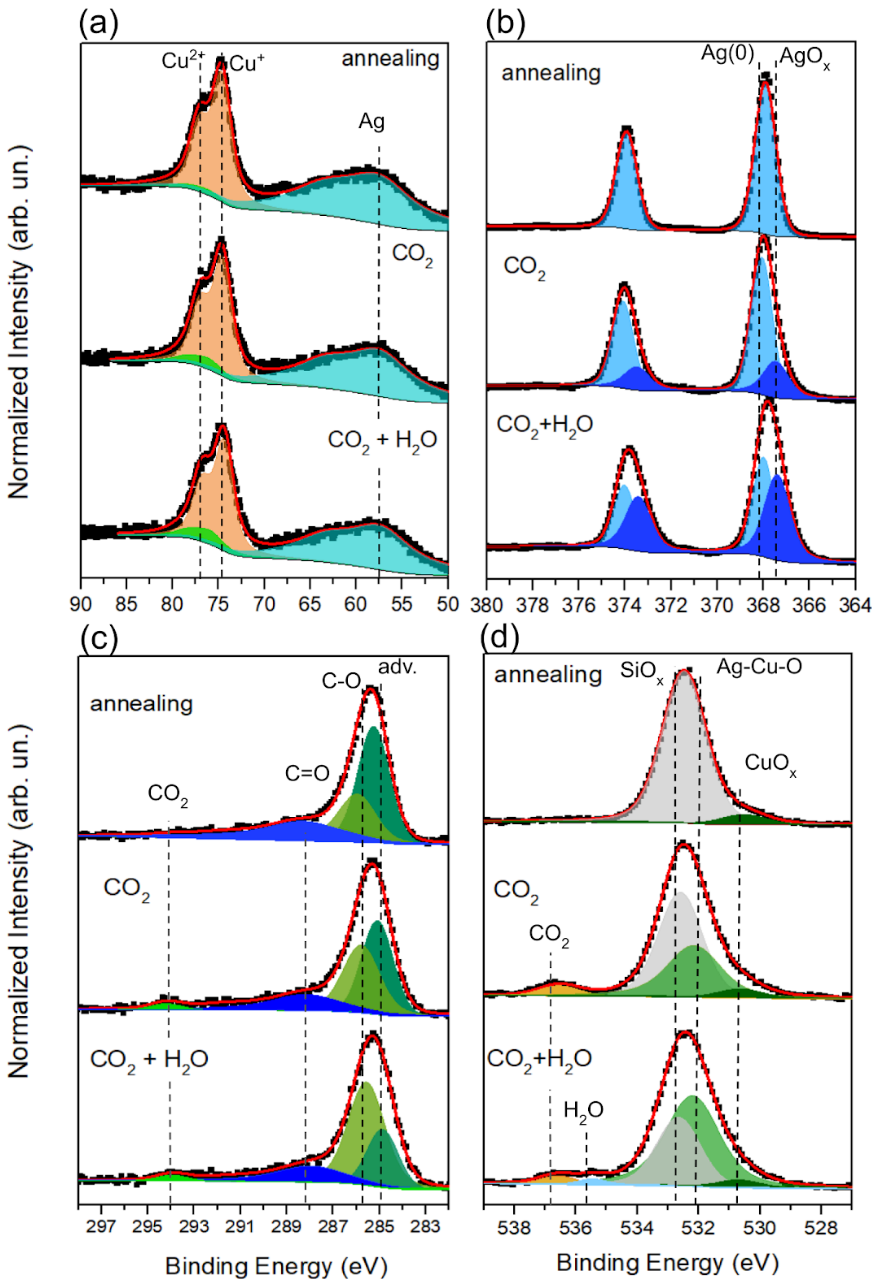

Figure S13 – AP-XPS spectra at (a) Cu 3p + Ag 4p, (b) Ag 3d, (c) C 1s, and (d) O 1s regions after annealing at 200 °C in 20 mTorr H_2, during exposure to 40 mTorr CO_2 and 40 mTorr CO_2 + 40 mTorr H_2O.

The AP-XPS measurements taken during exposure to the different conditions using photon energy of 1240 eV are shown in Figure S14. The fitting result shows similar components that were observed during measurements at beamline 9.3.2 using 695 eV photon energy. After annealing, the Cu^{2+} component decreases. The Ag 4p intensity increases relative to Cu 3p, showing that Ag is diffusing to the surface region. After laser irradiation, Ag 4p area decreases relative to Cu 3p, showing that the laser triggers Ag substitution by Cu.

Ag 3d regions could be fitted with a single component in almost all conditions. The energy resolution at beamline 11.0.2 was poorer than beamline 9.3.2 at the time of the measurement, which could explain the discrepancy. However, after laser irradiation it is still observable that two components can be used to fit the Ag 3d region, agreeing with the presence of an Ag oxide component, as observed before. The C 1s AP-XPS region shows the same components present in previous measurements. Laser irradiation promotes the growth of C-O relative to the adventitious component. The O 1s AP-XPS region shows that after annealing the Cu oxide component decreases, agreeing with previous measurements. After CO_2 + H_2O exposure, there is the appearance of the same intermediate component observed before, which is attributed to a Cu-Ag-O component. Another component in C 1s region due to the gas phase CO_2 is also present near 294 eV. After laser irradiation the Cu-Ag-O component decreases relative to the Cu oxide component, and a new component appears at higher binding energy. This is not related to Si because Si 2p area drastically decreases after laser irradiation. It is also not related to the formation of Cu or Ag oxides, as these are located in binding energy values[3-5]. This high binding energy component could be interpreted as physisorbed water, but it doesn't appear at the previous condition without light irradiation. Thus it is probably related to some adsorbed product from CO_2 reacting at the surface.

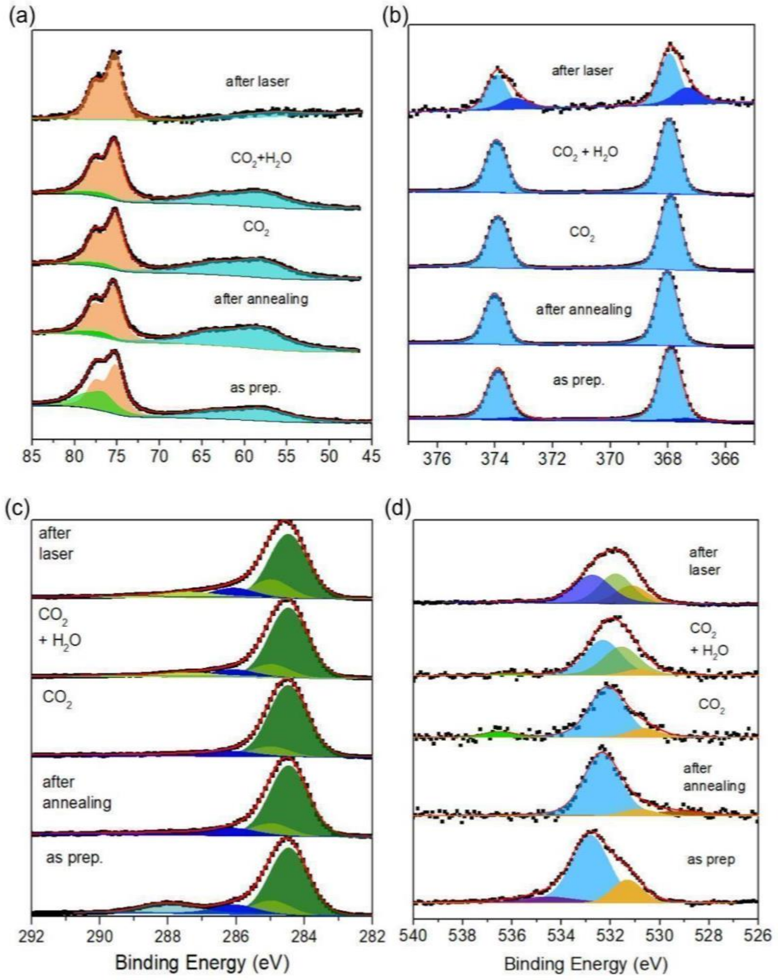

Figure S14: AP-XPS measurements at (a) Cu 3p + Ag 4p, (b) Ag 3d, (c) C 1s, and (d) O 1s electronic regions with photon energy of 1240 eV for the Ag-Cu/Si synthesized through thermal evaporation.

Figure S15 shows the O 1s AP-XPS spectra obtained during laser on and laser off conditions in beamline 11.0.2 during exposure to 40 mTorr CO_2 + 40 mTorr H_2O atmosphere after *in situ* XAS measurements. The fitting of the electronic regions takes into account three components. The low binding energy component is interpreted as CuO_x. The intermediate binding energy component is interpreted as Cu-Ag-O phase, and the high binding energy component is interpreted as the growth of a carbon component at the surface. The latter increases in relative intensity as the laser is turned on.

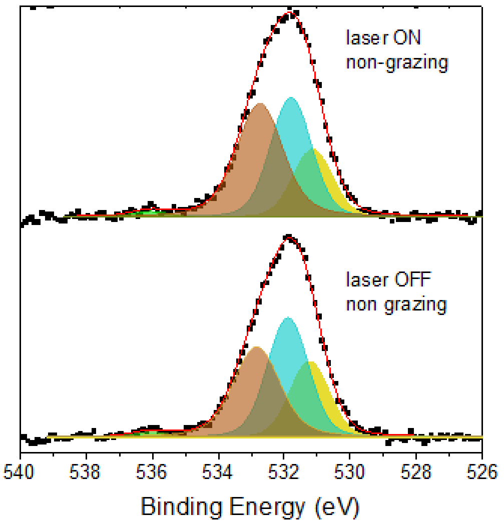

Figure S15: O 1s AP-XPS spectra measured during exposure to CO_2 + H_2O during laser on and after laser on condition.

Another experiment was performed in beamline 9.3.2 following the same procedures described previously. However, instead of irradiating the sample with the 532 nm as the last condition, the main chamber is brought to UHV again, and the sample is then exposed to an annealing procedure at 200 ºC in an 20 mTorr O_2 atmosphere. Figure S16 shows the comparison of the Cu 3p + Ag 4p and Ag 3d electronic regions between the sample exposed to CO_2 + H_2O atmosphere and to O_2 annealing. It can be observed that the overall shape of the Cu 3p region is not majorly modified. However the relative Cu/Ag intensity increases after annealing in O_2, showing that Ag diffuses to the core of the particles. The Ag 3d region shows that the overall shape of the electronic region is not affected by the procedure. This shows that the influence on the overall atomic concentration at the surface by annealing in an O_2 atmosphere under these circumstances is equivalent to irradiating the sample with a 532 nm laser.

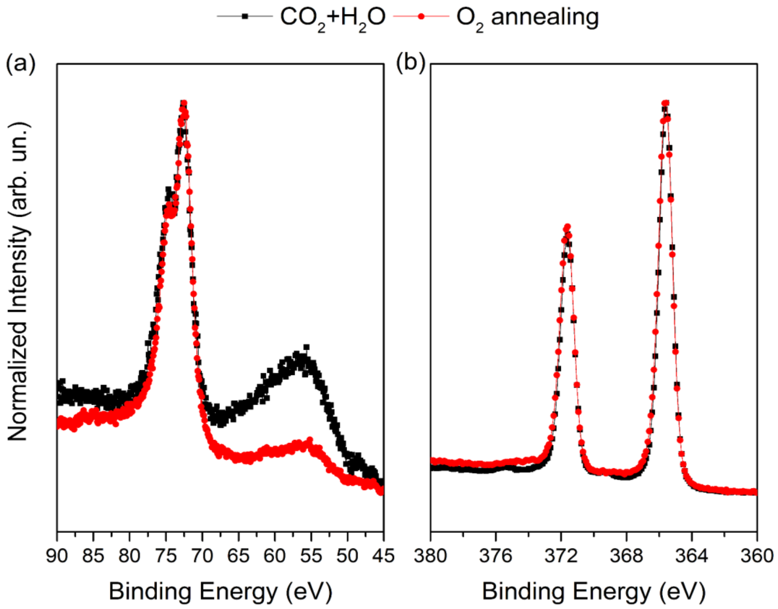

Figure S16: (a) Cu 3p + Ag 4p and (b) Ag 3d electronic regions during exposure to 40 mTorr CO_2 + 40 mTorr H_2O and after annealing in 20 mTorr O_2 atmosphere at 200 °C.

Figure S17 shows the total AP-XPS areas of each individual component obtained from fitting the C 1s electronic regions measured in 9.3.2 beamline. Total C 1s area only oscillates around 1% of its original value. However, adventitious and C=O component clearly drop in intensity with the different conditions, and C-O component rises instead.

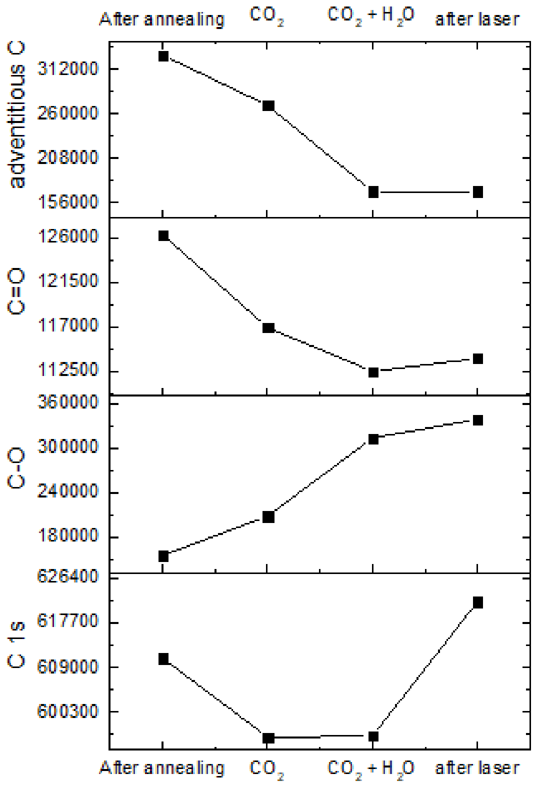

Figure S17: Areas of C 1s AP-XPS components obtained in different conditions during AP-XPS measurements at beamline 9.3.2 with 695 eV excitation energy.

The structure factor feature observed in the AP-GIXS measurements was fitted with a 2D gaussian peak-like function in a 500 pixel range to observe relative change in position (Figure S6). The peak center decreases in the q_y axis continuously from the as-prepared condition until the CO_2+H_2O condition. However, after 532 nm laser irradiation another substantial change is observed in the q_z position, indicating that the center of the nanoparticles is located in a higher position than before relative to the substrate.

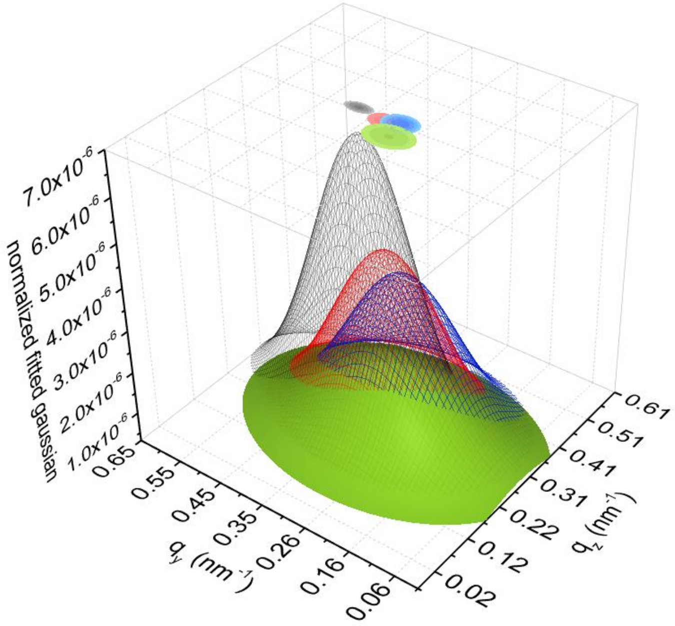

Figure S18: The surface plot showing four 2D gaussian functions fitted to the structure factor feature of the AP-GIXS measurement during CO_2RR. The black, red, and blue meshes represent the as prepared, annealing, and CO_2+H_2O conditions, while the green surface represents the measurement after 532 nm laser irradiation. The projections in the top plane show the center position and the relative spread of the gaussian functions between the conditions.

TEM images of Ag-Cu/Si nanoparticles were acquired at CMM BR-Sul-UFRGS using a JEOL JEM-1400 Flash operated at 120 kV. For the measurements, the Ag-Cu nanoparticles were shaved from the Ag-Cu/Si wafer, dispersed in deionized water and left in the ultrasound for 15-20 min. Figure S19 shows a typical TEM image of the Ag-Cu nanoparticles (a) before and (b) after CO_2RR, where the shape change is clearly observed.

(a) (b)

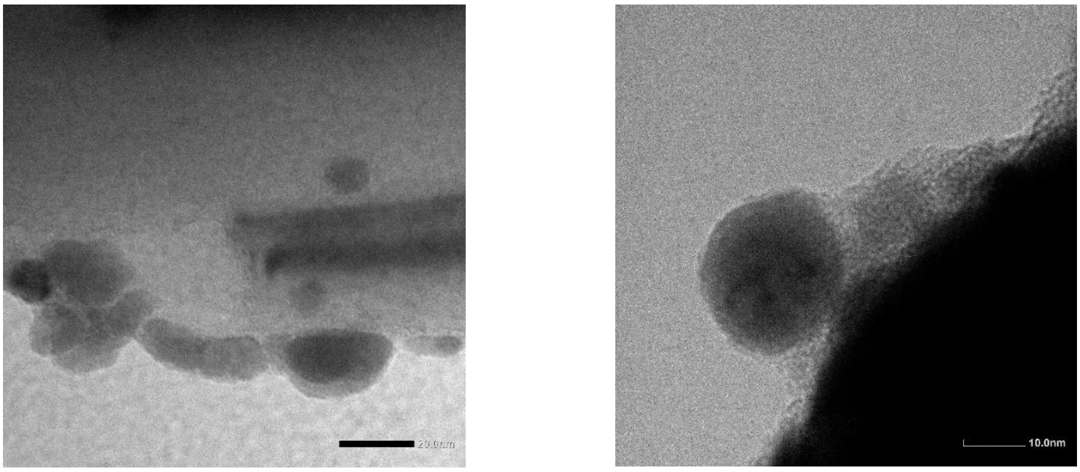

Figure S19 – Typical TEM image of the Ag-Cu nanoparticles after removal from Si wafer (a) before and (b) after photocatalytical CO_2RR.

Figure S20 shows the area of the Si 2p AP-XPS obtained with 1240 eV at beamline 11.0.2 component normalized to the area of C 1s + O 1s + Ag 3d + Cu 3p regions using the survey spectra (Figure S20). It can be observed that the intensity of Si 2p decreases mainly after annealing, which comes from the Ag-Cu spreading over the surface. The same occurs after laser irradiation, but in this case there is the growth of a thick C layer at the sample surface.

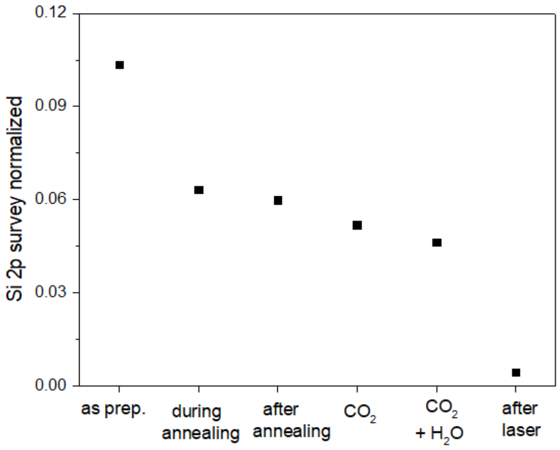

Figure S20: Normalized Si 2p AP-XPS area by the C 1s +, O 1s, + Ag 3d + Cu 3p area as a function of the treatment employed.

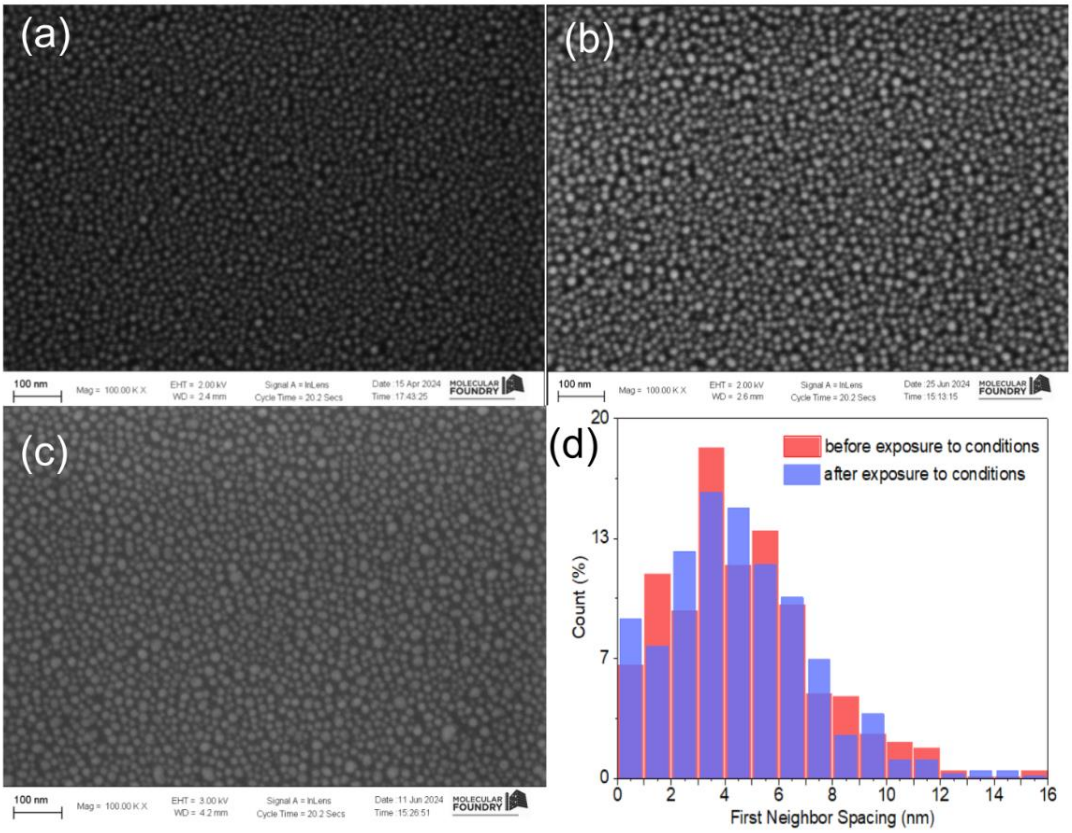

Figure S21: Scanning Electron Microscopy images of (a) as prepared sample, (b) after exposure to conditions at 9.3.2 beamtime, and (c) after exposure to conditions at 11.0.2 beamtime. (d) First neighbor spacing histogram taken before and after exposure to conditions at 11.0.2 beamline.

Figure S22 shows the fitting of the out-of-plane linecuts taken at q_y = -0.3 nm^{-1}. Choosing where the linecut is taken for usual SAXS analysis must follow some important considerations. First, near $q_y = 0$ nm^{-1} the scattering is affected significantly by the reflectivity signal of the substrate. At q_y = -1.0 nm^{-1}, the signal of the Yoneda line is much stronger than the signal of the particles. Also higher absolute values of q_y present increased warping due to the detection geometry. The existence of an in-plane structure factor originating from the particles around q_y = -0.3 nm^{-1} strengthens the scattering signal at the out-of-plane direction, thus it was chosen for further analysis. The fitting procedure takes into account a single form factor. It is the one of a sphere but taking into consideration that the scattering density can be varied along the radial distance inside the sphere. The variation is taken into account by defining the following scattering density

$$\eta(r) \; = \; \eta_0 \, (1 - (r/R)^2 \,)^{\alpha/2-2}$$

Where R is the average external radius and η_0 the scattering contrast. By varying the α parameter, with lower limit equal to 2, it is possible to either obtain a sphere in which the scattering density of the core is higher than the shell ($\alpha > 4$), equal to the shell ($\alpha = 4$), or lower than the shell ($\alpha < 4$).

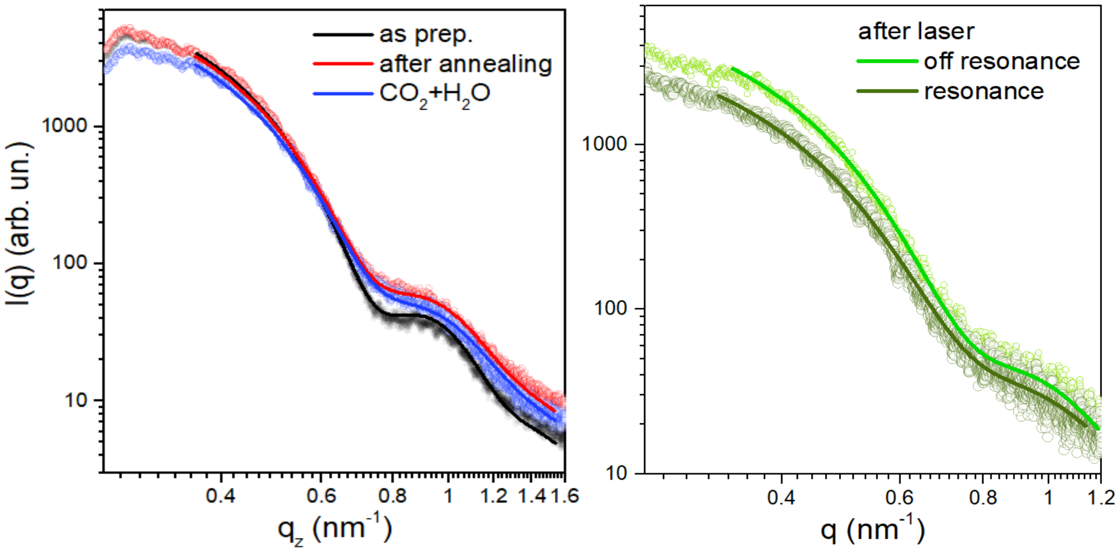

Figure S22: Fitting of linecuts taken at q_y = -0.3 nm^{-1} with the Boucher sphere model.

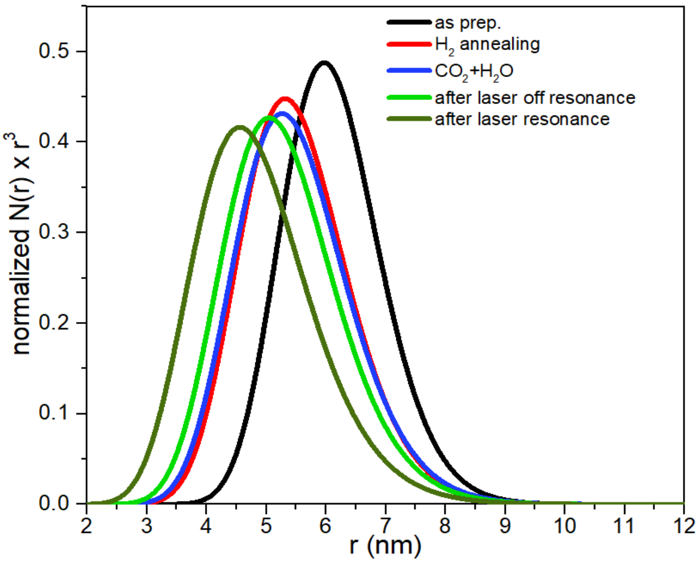

Figure S23: Size distribution obtained after the fitting procedure.

Figure S24 compares the information obtained through the Boucher model and the BornAgain simulation for the as prepared sample.

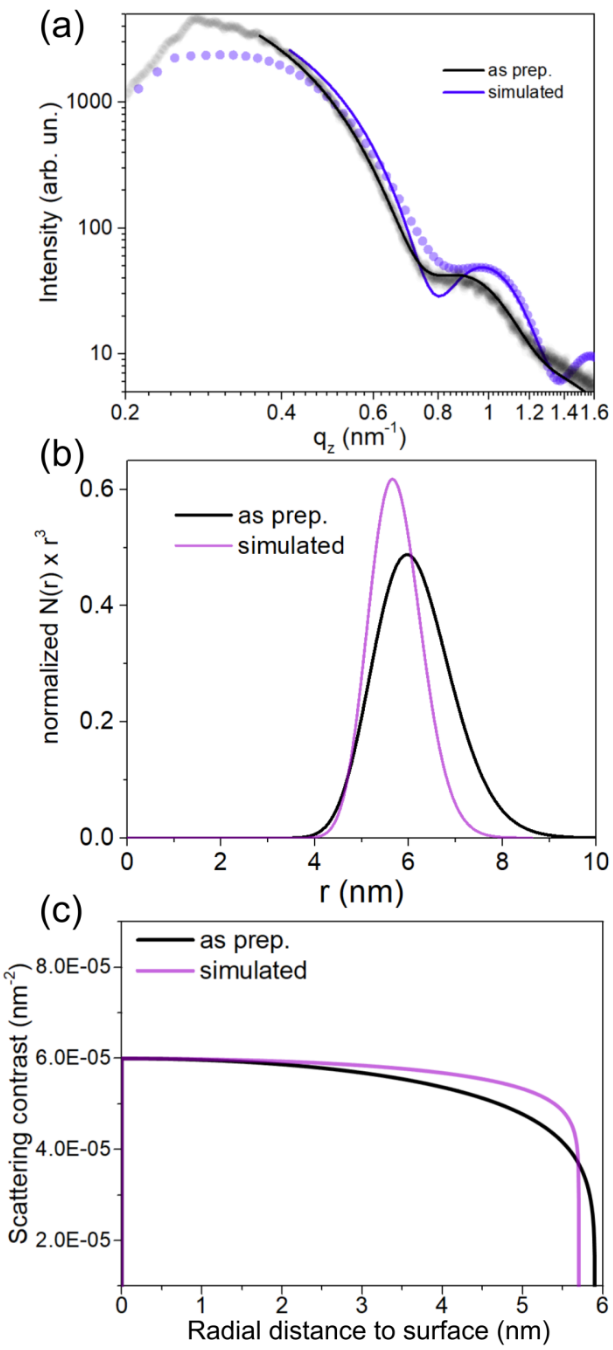

Figure S24: (a) Fitting of the BornAgain simulated linecuts taken at q_y = -0.3 nm^{-1} with the Boucher sphere model, (b) comparison of size distributions, and (c) scattering contrast as a function of the radial distance to the surface of the average-size particle.

The final morphology and main features of the nanoparticles after thermal annealing at varied temperatures were analyzed by MD simulations by visual inspection, and some quantitative metrics were computed for further assessment as explained in the Methods

section of the main paper. To evaluate their relative stability, we compute the formation energy

$$E_f = \frac{(E_{NP} - N_{Ag}E_{Ag} - N_{Cu}E_{Cu})}{N_{NP}}$$

where E_{NP} and N_{NP} are the total energy and total number of atoms in the nanoparticle, E_{Ag} and E_{Cu} the energy of Ag and Cu atoms in a bulk phase, and N_{Ag} and N_{Cu} are the number of Ag and Cu atoms in the nanoparticles. To assess the diffusion of Ag to the nanoparticle surface, we computed the stoichiometry of the surface (number of Ag atoms on the surface/total number of atoms on the surface). The characteristic dimensions along the three axes and eccentricity of ellipsoids were also evaluated.

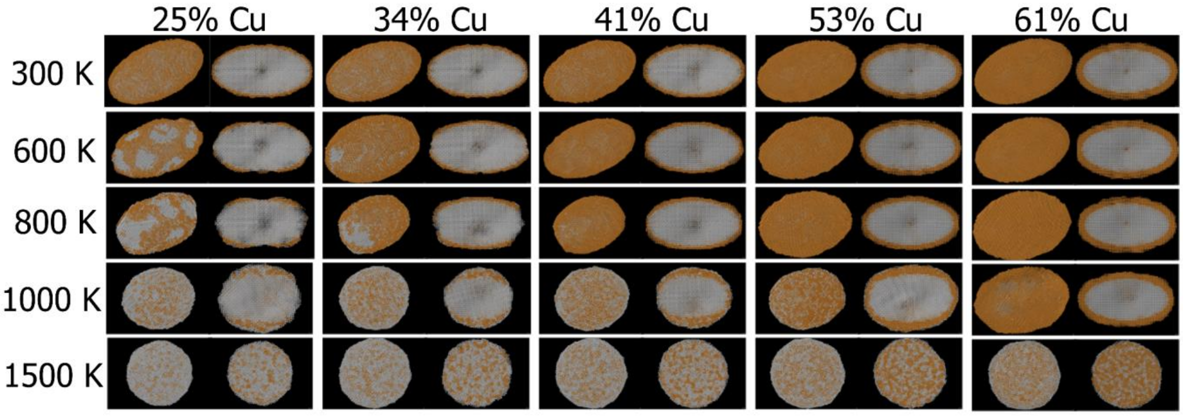

Figure S25: Atomic configurations obtained for initially elongated Ag-Cu nanoparticles with different Cu/Ag ratios after annealing in different temperatures.

Figure S26 shows the specific energies obtained after simulations. It is interesting to notice that at 1500 K the formation of multiple interfaces between Ag and Cu happens inside the nanoparticle, which increases the specific energy of the system.

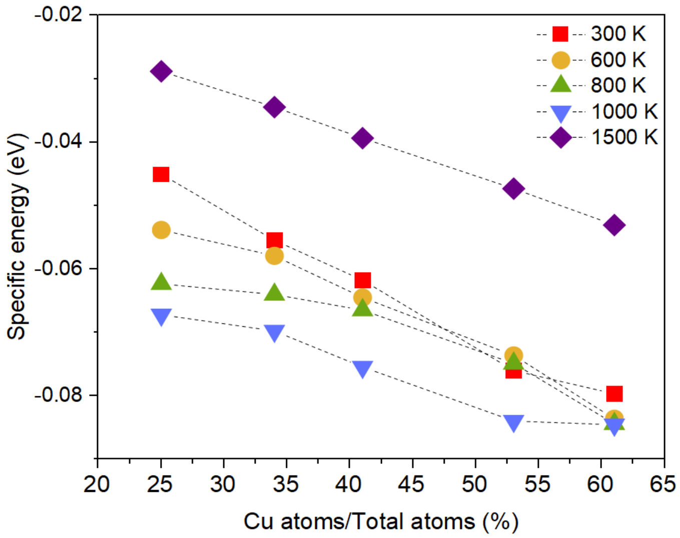

Figure S26: Specific energies of the ellipsoidal Ag-Cu nanoparticles as a function of composition and annealing temperature.

Results for MD simulation of thermal annealing for initially elongated pure Ag nanoparticles are seen in Figure S27. It is observed that higher temperatures than those seen for Ag-Cu nanoparticles are required to achieve significant changes in their characteristic dimensions.

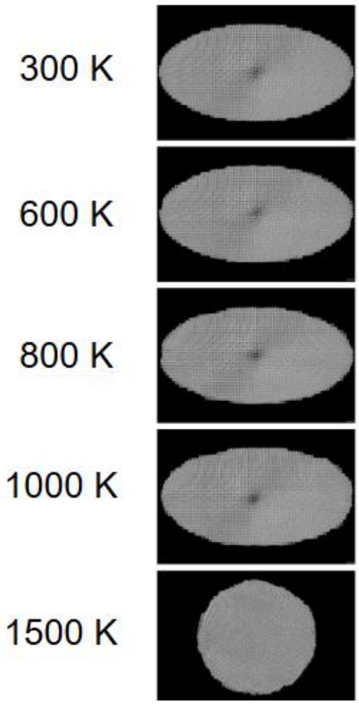

Figure S27: Atomic configurations obtained for elongated Ag nanoparticles after annealing in different temperatures.

Additional information

Original raw data image of AP-GIXS measurements in the as prepared sample is shown in Figure S28. It can be seen that it is warped due to the particular measurement geometry and possible tilting of the sample. Points are chosen on the image along the main maxima on both left and right parts. The x and y pixels on left part are fitted using a Boltzmann function, while the ones on the right part are fitted using a Lorentz function. Then, the maxima along y-axis are required to be placed along the same lowest x-axis maxima. The ones between the left and right functions are interpolated. Every constant x-axis line receives the same transposition. After transposition, the image is smoothed through a 2D gaussian filter with a 30 pixel standard deviation. This is done in order to reduce the wavelike features in the image that exist because of signal from the SiN_x window, and subsequent stitching of the multiple frames captured at different angles to form the whole image.

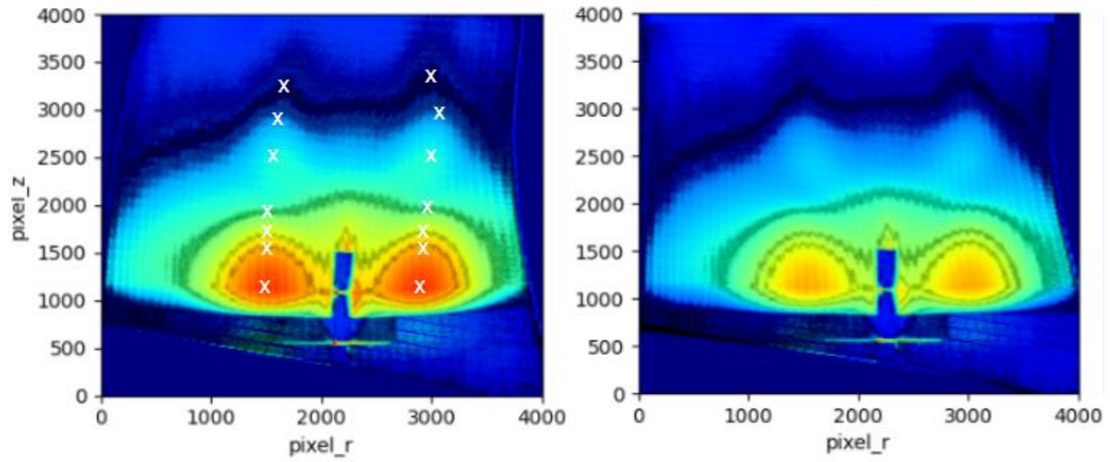

Figure S28: (a) Original raw image of the as-prepared condition, white “x” mark some of the maxima points chosen. (b) Image result after transposition treatment. The image gets visibly less warped.

Figure S29 shows the in-plane linecuts taken at q_z = 0.35 nm^{-1}. The curves shift towards lower q values with every subsequent condition, evidencing that the samples’ lateral size is increasing. After laser irradiation, and at resonant Cu L edge condition, the maxima related to the structure factor decreases.

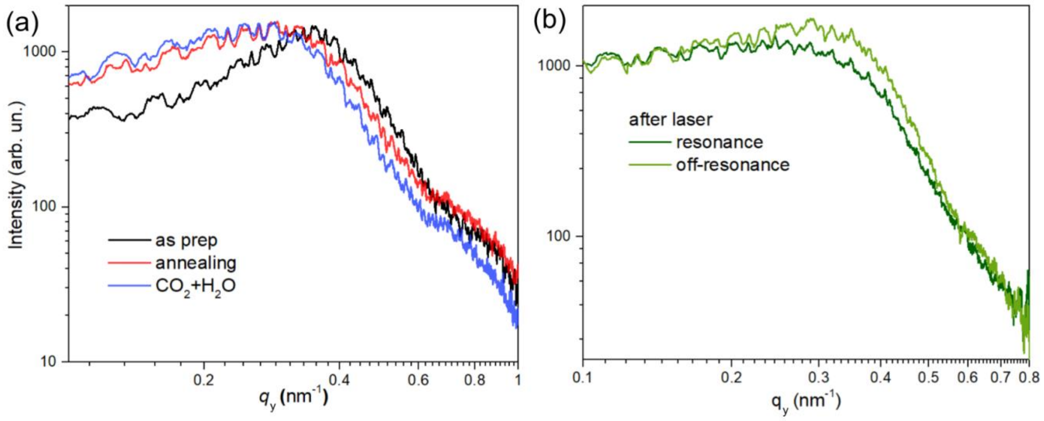

Figure S29: Comparison of in-plane linecuts taken at 0.35 nm^{-1} during CO_2RR.

Figure S30 shows the comparison between the linecuts of the as-prepared sample with the linecuts taken from the BornAgain simulation. Apart from the q = 1 nm^{-1} linecuts, that are heavily affected by warping due to the detection geometry, all data show excellent agreement.

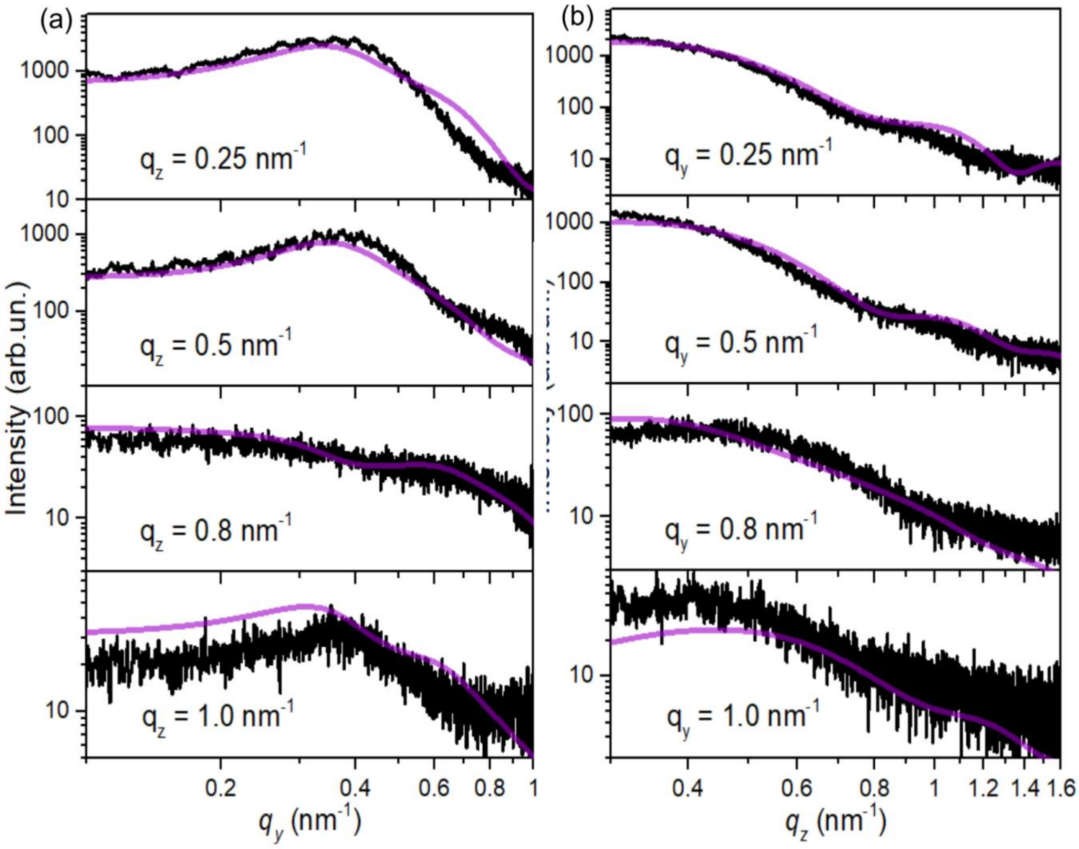

Figure S30: Comparison between linecuts of the as prepared sample (black lines) and the BornAgain simulation (purple lines) in (a) in-plane direction, and (b) out-of-plane direction.

We also analyzed spherical particles with diameters within 3-7 nm and a composition of 25% Ag, and with a diameter of 3 nm and compositions of 25, 50 and 75% Ag. Examples of initial configurations of the nanoparticles with varied core-shell morphologies, diameters and compositions are given in Figures S31 and S32. Similar trends to those discussed above and in the main text for ellipsoidal nanoparticles are seen, regarding the tendency of Ag diffusion toward the surface at higher temperatures. It is interesting to notice that in small temperatures and low Cu concentration the Cu atoms aggregate in clusters on top of the edges of the Ag nanoparticles.

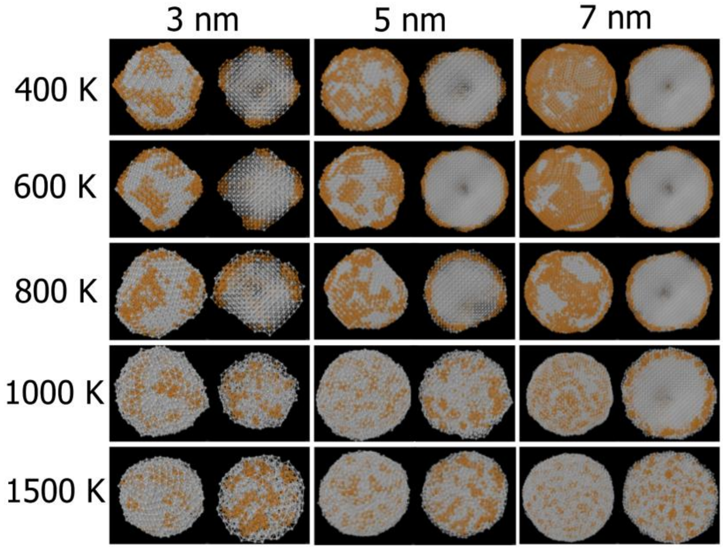

Figure S31: Atomic configurations obtained for spherical 25% Cu - 75% Ag nanoparticles with different sizes after annealing in different temperatures.

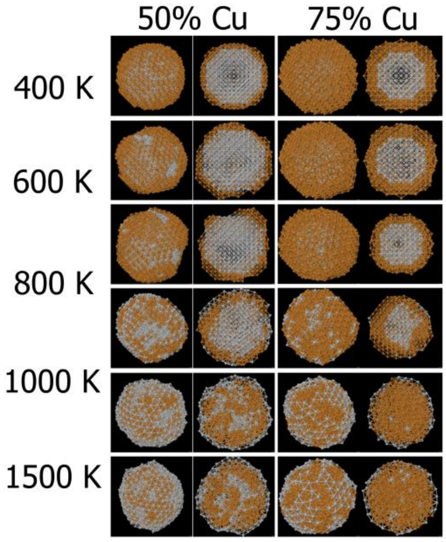

Figure S32: Atomic configurations obtained for spherical Ag-Cu nanoparticles with 3 nm size and different Cu/Ag rations after annealing in different temperatures.

References

1. Nečas, D. & Klapetek, P., Gwyddion: an open-source software for SPM data analysis. Open Physics 10, 181–188 (2012).
2. Matte, L. P.; Khan. W.; Thill, A. S.; Escudero, C.; Poletto, F.; Bernardi, F. Controllable morphology of Pd nanostructures: from nanoparticles to nanofoams. Materials Research Express 11, 105010 (2024).

3 Hoflund, G. B., Weaver, J. F. & Epling, W. S., Ag_2O XPS spectra. Surface Science Spectra 3, 157–162 (1994).

4. Hoflund, G. B., Weaver, J. F. & Epling, W. S., AgO XPS spectra. Surface Science Spectra 3, 163–168 (1994).
5. Van Veenendaal, M. A. & Sawatzky, G. A., Intersite interactions in Cu L-edge XPS, XAS, and XES of doped and undoped Cu compounds. Physical Review B 49, 3473 (1994).
